# Heat shock protein A2 is a novel extracellular vesicle-associated protein

**DOI:** 10.1038/s41598-023-31962-5

**Published:** 2023-03-23

**Authors:** Damian Robert Sojka, Agata Abramowicz, Małgorzata Adamiec-Organiściok, Elżbieta Karnas, Łukasz Mielańczyk, Daria Kania, Sławomir Blamek, Ewa Telka, Dorota Scieglinska

**Affiliations:** 1 Maria Sklodowska-Curie National Research Institute of Oncology, Gliwice Branch, Wybrzeże Armii Krajowej 15, 44-102 Gliwice, Poland; 2 Department of Systems Biology and Engineering, Silesian University of Technology, Institute of Automatic Control, Akademicka 16, 44-100 Gliwice, Poland; 3 Department of Cell Biology, Faculty of Biochemistry, Biophysics and Biotechnology, Jagiellonian University, Gronostajowa 7 St., 30-387 Kraków, Poland; 4 Department of Histology and Cell Pathology, Faculty of Medical Sciences in Zabrze, Medical University of Silesia, Katowice, Poland

**Keywords:** Cancer, Cell biology, Extracellular signalling molecules, Stress signalling, Molecular biology, Chaperones

## Abstract

70-kDa Heat Shock Proteins (HSPA/HSP70) are chaperones playing a central role in the proteostasis control mechanisms. Their basal expression can be highly elevated as an adaptive response to environmental and pathophysiological stress conditions. HSPA2, one of poorly characterised chaperones of the HSPA/HSP70 family, has recently emerged as epithelial cells differentiation-related factor. It is also commonly expressed in cancer cells, where its functional significance remains unclear. Previously, we have found that proteotoxic stress provokes a decrease in HSPA2 levels in cancer cells. In the present study we found that proteasome inhibition-related loss of HSPA2 from cancer cells neither is related to a block in the gene transcription nor does it relate to increased autophagy-mediated disposals of the protein. Proteotoxic stress stimulated extracellular release of HSPA2 in extracellular vesicles (EVs). Interestingly, EVs containing HSPA2 are also released by non-stressed cancer and normal cells. In human urinary EVs levels of HSPA2 were correlated with the levels of TSG101, one of the main EVs markers. We conclude that HSPA2 may constitute basic components of EVs. Nevertheless, its specific role in EVs and cell-to-cell communication requires further investigation.

## Introduction

The Heat Shock Protein A (HSPA) family comprises 12 highly conserved molecular chaperone proteins of 70-kDa molecular weight. Structurally, HSPAs consist of two major domains: the N-terminal nucleotide-binding domain (NBD) with ATPase activity, joined by a flexible linker to the C-terminal substrate binding domain (SBD)^1^. The main physiological functions of proteins from the *HSPA* family are folding of nascent and denatured proteins to the native state, refolding of aggregated proteins, and targeting for degradation of irreparably damaged proteins by ubiquitin-proteasome system or via the process of chaperone-mediated autophagy (CMA). HSPAs are considered guardians of cell’s proteome quality, consequently, they are critically important for cell’s ability to face various types of environmental and pathophysiological stresses.

HSPAs are considered as intracellular proteins. Five of the most homologous HSPAs (HSPA1, HSPA2, HSPA6, HSPA8, HSPA1L) can shuttle between the cytoplasm and the nucleus. Certain HSPA paralogs localise preferentially to certain cellular compartments (mitochondria, endoplasmic reticulum, Golgi apparatus, nucleus). Nonetheless, HSPAs can be present at the luminal side of the endosomal-lysosomal system and on the plasma membrane. The membranous location of HSPAs is specific to cancer cells; it is believed that normal healthy cells do not bear HSPA on their surface^2^. Albeit a classical transmembrane sequence is missing in HSPAs, in vitro studies revealed that different potential mechanisms are implicated in crossing of HSPAs through the plasma membrane or the endosomal-lysosomal compartment^3–5^. Importantly, in cancer cells the affinity of HSPAs (HSP70) to membranes is linked with cytoprotection due to the ability to block the lysosome-dependent cell death^6^. Moreover, membranous location of HSPA (HSP70) yields an effective anticancer immune response, thereby offering prospective opportunities for cancer treatment^7,8^.

Cells upon environmental and pathophysiological stress can also release HSPAs to extracellular milieu in a free soluble form or in association with small extracellular vesicles (EVs). EVs, one of the key players in the intercellular communication, are released by all types of cells^9^ and their cargo consists of the characteristic set of proteins, including CD63, CD9, CD81, TSG101 or Alix and also of certain proteins specific to parental cells^10,11^. These multifunctional lipid-bilayer enclosed structures are able to transfer information by interaction with receptors, direct fusion with the plasma membrane, or by endocytosis^12^. The diverse cargo includes proteins, RNAs as well as various lipids and metabolites, that can be successfully taken up by recipient cells via clathrin-, lipid-raft- or caveolin-mediated endocytosis as well as by phagocytosis or micropinocytosis^9,13^. The internalised molecules may be processed through the typical endosomal pathway and degraded in lysosomes, or they can avoid degradation and interact with intracellular targets significantly affecting cellular functions/signaling^13,14^. One of the well-characterised functions of EVs is their involvement in response to cellular stresses^15^. This feature of EVs is extensively studied due to its role for the development of therapy resistance^16^. Importantly, HSPAs and other HSPs (e.g. HSPC2/HSP90AB1 or HSP27) have been indicated as the constitutive EV proteins^17–21^. There is accumulating evidence that EVs are an important alternative secretory pathway of HSPA both in physiological and stress conditions^22,23^. Moreover, despite the common presence of HSPAs in EVs, the membrane localisation of stress-induced HSPA/HSP70 is specific only to tumour-derived EVs and was proposed as a potential diagnostic or treatment biomarker^24,25^. However current understanding of HSPAs significance in EVs-mediated communication is far from being satisfactory.

HSPA2, one of stress-non-inducible members of the HSPA family, was originally considered as testis-specific. However this protein is also present in certain human somatic tissues, including multilayered epithelia^26^. The role of HSPA2 for male fertility is well characterised^27^, nevertheless, its importance beyond its function in spermatogenic cells is poorly understood. We have recently found that HSPA2 supports high clonogenic potential of normal cells derived from human bronchial epithelium or epidermis^28,29^. HSPA2 is also ubiquitous in various types of cancer cells^30–32^, but in vitro studies put under question its importance for cytoprotective or cancer-promoting mechanisms in lung, breast, or cervical cancer cells^29,33^. Moreover, HSPA2 is lost from cancer cells upon proteotoxic stress conditions induced by different proteasome inhibitors^33–35^ that stimulate the phosphorylation of heat shock factor 1 (HSF1), the main activator of HSPA genes expression^36^. Proteasome inhibition led to a decrease in cellular level of HSPA2 and a massive accumulation of HSPA1 and HSPA6, which are encoded by the canonical HSF1-dependent genes. Here we aimed at examining this phenomenon to search mechanisms that control intracellular levels of HSPA chaperones. We have found that HSPA2 is indeed a component of EVs released from both cancer and normal cells.

## Results

### Proteasome inhibition downregulated HSPA2 expression at the protein level

**Figure 1:**
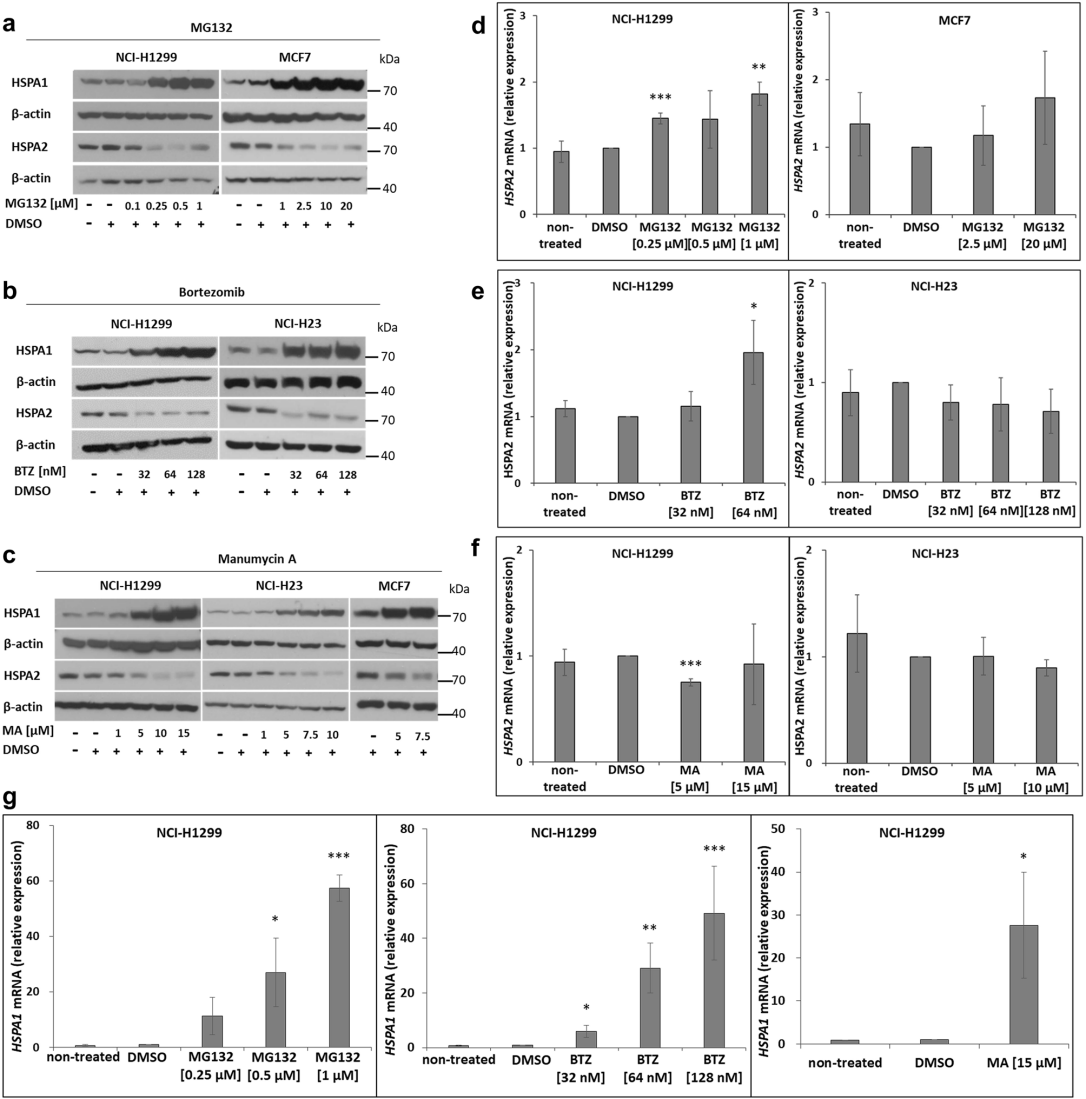
Changes in HSPA1 and HSPA2 expression in cells exposed to proteasome inhibitors. (**a**–**c**) Levels of HSPA1 and HSPA2 proteins after treatment with MG132 (**a**), Bortezomib (BTZ) (**b**), or Manumycin A (MA) (**c**). Representative immunoblots are provided (*n* ≥ 3). β-actin was used as a protein loading control. Prior to incubation with primary antibody membranes were cut according to 55 kDa molecular ladder band. Chemiluminescent signal was detected on X-ray film. Original autoradiograms/immunoblots are presented in Fig. S2. (**d**–**g**) Effects of proteasome inhibitors (24 h) on the *HSPA2* (**d**–**f**) and *HSPA1* (**g**) mRNA levels. Results of RT‐qPCR analysis showed as mean ± SD from at least three independent experiments, each in three technical replicas. *RPL13A, TMEM43, and B2M* were used as the reference genes (Table S1). Statistical significance was determined by the two-tailed t-test performed in regard to cells exposed to DMSO solvent only. *P < 0.05; **P < 0.01; ***P < 0.001.

We compared the effect of proteasome inhibition on HSPA2 expression in NCI-H1299, NCI-H23 and MCF7 cancer cell lines derived from non-small cell lung cancer (NSCLC) and breast cancer, respectively. The cells were treated (24 h) with MG132 (Fig. 1a) and bortezomib (BTZ) (Fig. 1b), two well-studied reversible proteasome inhibitors, and manumycin A (MA) (Fig. 1c), that is known to have an inhibitory effect on proteasome function^37,38^. Each of the compounds caused a significant dose-dependent reduction in the protein level of HSPA2 (Fig. 1a–c) that was not accompanied by a noticeable decrease in the mRNA expression levels (Fig. 1d–f). As expected, the proteasome inhibition resulted in a massive increase in the *HSPA1A/B* gene expression, a major stress-inducible member of HSPA family and stress marker, both at the protein (Fig. 1a–c) and mRNA levels (Fig. 1g).

### Autophagy is not involved in the HSPA2 degradation upon proteasome inhibition

Subsequently, we examined whether the drop in the HSPA2 protein level upon the arrest of proteasomal protein degradation pathways was dependent on autophagy-related proteolysis pathways. We found (using KFERQ finder V0.8 program) that HSPA2 bears two chaperone-mediated autophagy (CMA) recognition sequences (KFERQ-like motifs). CMA is a targeted, lysosome-dependent proteolytic system that eliminates cytoplasmic proteins containing KFERQ motif. Therefore, we used bafilomycin A1 (BAF), a vacuolar H + ATPase (V-ATPase) and autophagy inhibitor, which is a potent blocker of total lysosomal proteolysis^39^. Given that HSPA8 (HSC70) is a crucial player in CMA^40^, we also used VER-155008 (VER) at non-toxic concentration to inhibit HSPA/HSPA8 activity in BTZ-treated cells^33,41^. Chloroquine (CHQ), that is known to prevent the binding of autophagosomes to lysosomes, was used as an additional inhibitor of autophagy-dependent proteolysis.

**Figure 2:**
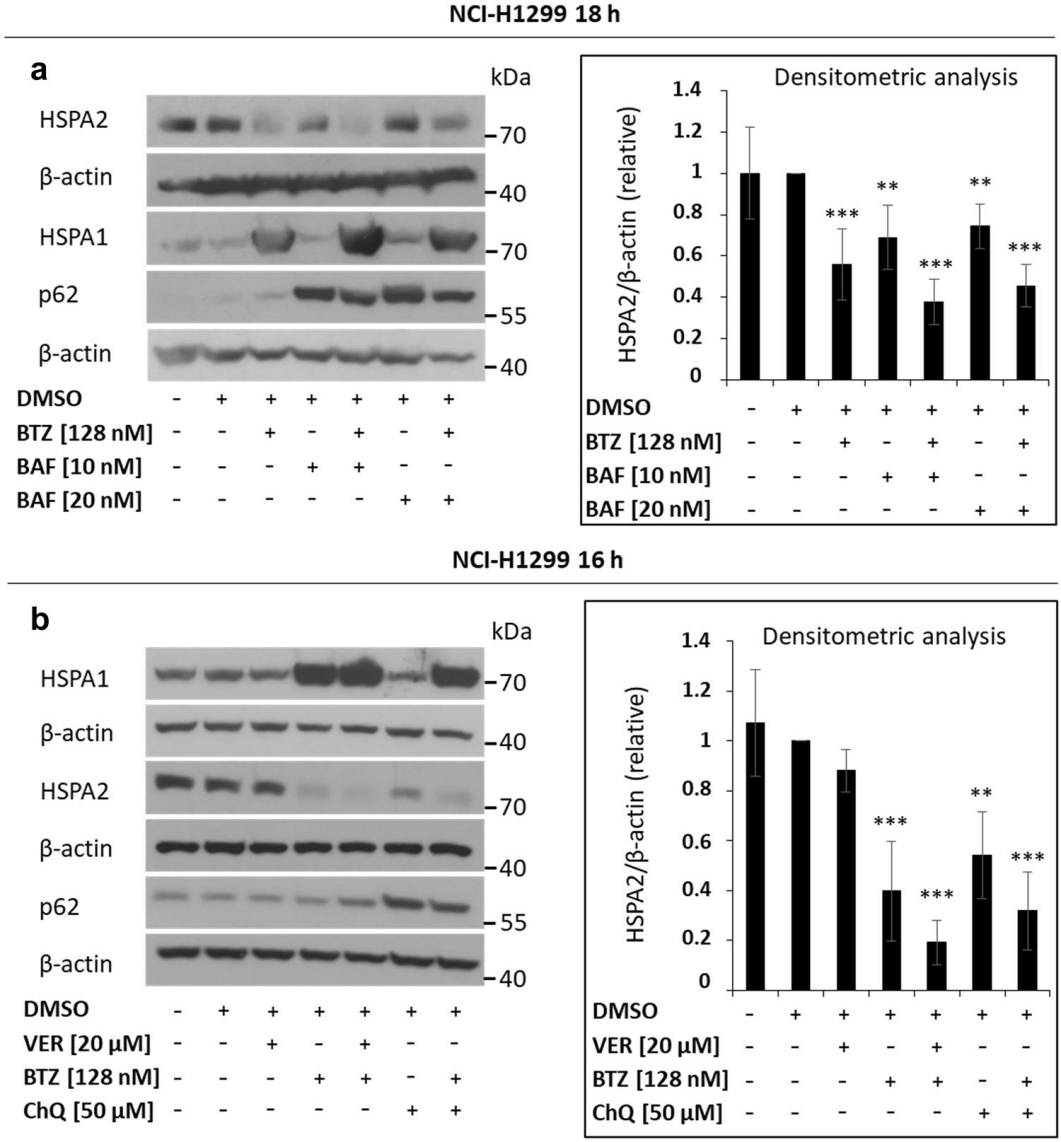
Autophagy inhibition does not prevent proteasome inhibition-related reduction in intracellular levels of HSPA2. (**a**) Effects of the single or combined treatment with bafilomycin A (BAF) and bortezomib (BTZ) on the HSPA1, HSPA2, and p62 protein levels. (**b**) Effects of a single or combined treatment (16 h) with VER-155008 (VER), BTZ, or chloroquine (ChQ) on the protein levels of HSPA1, HSPA2 and p62. In either experiment, HSPA1 and p62 were used as a BTZ or ChQ/BAF treatment control, respectively. β-actin was used as a protein loading control. Prior to incubation with primary antibodies membranes were cut according to the molecular ladder band (55 kDa). For chemiluminescent signal detection X-ray film was used. Original autoradiograms/immunoblots are presented in Fig. S3. Graphs on the right side show results of densitometric analysis of immunoblots representative for HSPA2 expression (mean ± SD; n ≥ 4). The protein level is presented relative to β-actin. Statistical significance was determined by the two-tailed t-test performed in regard to cells exposed to DMSO solvent only. *p < 0.05; **p < 0.01; ***p < 0.001.

Accumulation of HSPA1 confirmed proteasome inhibition in BTZ-treated NCI-H1299 cells (Fig. 2a,b). Co-treatment with BAF and BTZ (Fig. 2a) as well as with ChQ and BTZ (Fig. 2b) led to inhibition of autophagy-dependent proteolysis as proved by elevation of p62, a long-lived protein and approved autophagy marker. VER alone (Fig. 2b) did not affect the HSPA2 levels, whereas BAF (Fig. 2a) or CHQ (Fig. 2b) alone lead to reduction of the HSPA2 level. However, neither combination of BTZ with BAF (Fig. 2a), nor with CHQ (Fig. 2b) nor with VER (Fig. 2b) reversed the drop in HSPA2 level caused by BTZ. Altogether, our results suggest that a massive decrease of HSPA2 upon proteasome inhibition is not due to increased autophagy-dependent proteolysis of HSPA2.

### HSPA2 is secreted to extracellular environment

**Figure 3:**
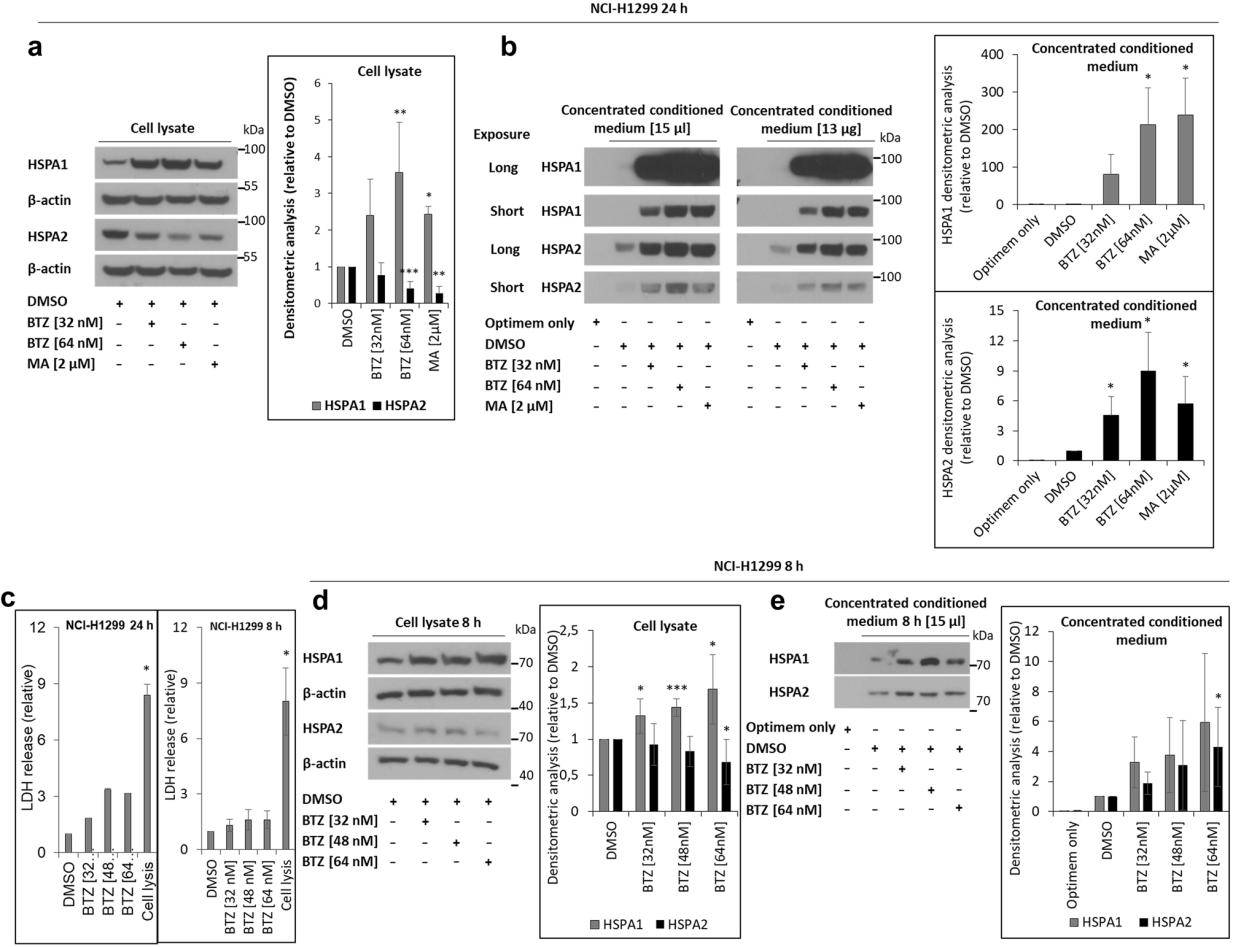
Proteasome inhibition stimulates extracellular release of the HSPA2 and HSPA1 proteins. (**a**, **d**) Intracellular and (**b**, **e**) extracellular levels of HSPA1 and HSPA2 in cells exposed to bortezomib (BTZ) or manumycin A (MA). Extracellular HSPAs were detected in concentrated conditioned FBS-free media (Opti-MEM™). Samples were prepared in relation to the sample volume (µl) or protein concentration (µg; in (B) only); concentrated Opti-MEM™ only sample was tested as a negative control. Prior to incubation with primary antibody membranes were cut according to the 55 kDa molecular ladder band. X-ray film was used for chemiluminescent signal detection. Original autoradiograms/immunoblots are presented in Fig. S4. Graphs in (**a**, **b**, **d**, **e**) show results of densitometric analysis of immunoblots for HSPA1 and HSPA2 detection (mean ± SD; n ≥ 3)**.** In (**b**) ‘volume’ group was quantified. (**c**) Results of lactate dehydrogenase (LDH) release detection assay are expressed in relation to the DMSO-treated control cells (mean values ± SD; n = 3, each in duplicate). Statistical significance was determined using the two-tailed t-test. *p < 0.05; **p < 0.01; ***p < 0.001.

HSPs, including HSPA1, can be secreted from the cells in a free protein form or as EVs cargo^42^. Therefore, we investigated the presence of HSPA2 in conditioned media from BTZ- or MA-treated (24 h) NCI-H1299 cells. We found that the drug-induced decrease of intracellular HSPA2 (Fig. 3a) was paralleled by an increase in its amount in extracellular space (Fig. 3b).The extracellular HSPA2 was also easily detectable in media from non-treated cells (Fig. 3b). HSPA1, in turn, was barely detectable outside the non-stressed cells, but its intracellular and extracellular levels raised after the treatment (Fig. 3b). It is noteworthy that the pattern of HSPA1 and HSPA2 detection by Western blot (WB) was not influenced by protein sample preparation method (relatively to its volume or protein concentration) (Fig. 3b).

Increased release of lactate dehydrogenase (LDH) indicated that a 24 h-long treatment with MA or BTZ was toxic to the cells (Fig. 3c). Thus, to exclude the possibility that the release of extracellular HSPAs reflects their passive leak from dying cells, we performed a similar analysis but with endpoint that preceded manifestation of BTZ-induced toxicity (minimal LDH leakage, Fig. 3c). After shorter (8 h-long) BTZ treatment, intracellular levels of HSPA1 were slightly higher, whereas the HSPA2 ones were lower in comparison to non-treated cells (Fig. 3d). In parallel, levels of extracellular HSPA1 and HSPA2 were higher in media from BTZ-treated than from non-treated cells (Fig. 3e). Altogether, these results indicated that stress conditions augment the release of HSPA2 into extracellular space.

### Extracellular HSPA2 is related to small extracellular vesicles (EVs)

**Figure 4:**
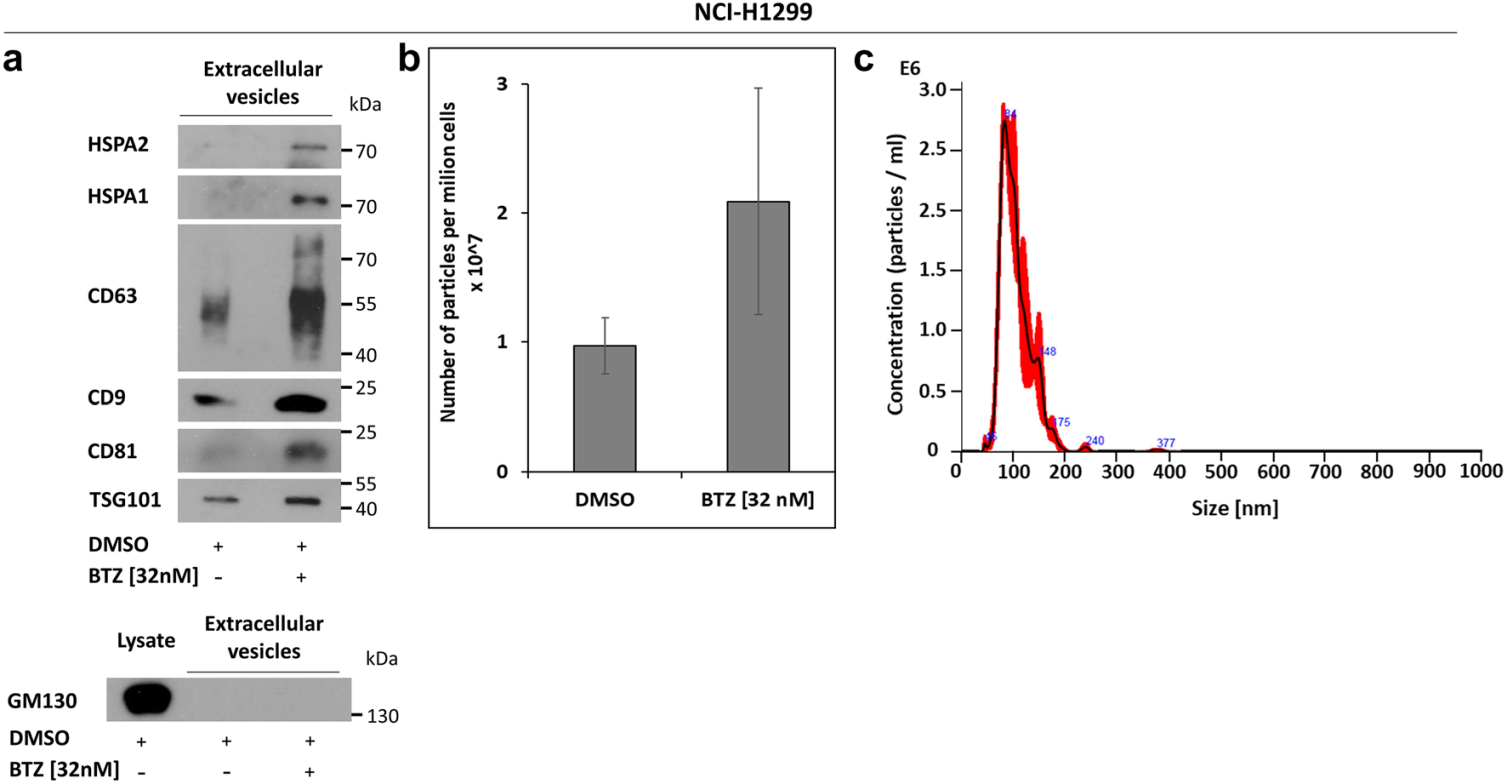
HSPA2 is present in SEC-purified small extracellular vesicles (EVs) from NCI-H1299 cells. (**a**) Levels of HSPA2, HSPA1, and EVs marker in EVs derived from non-treated and BTZ-treated (32 nM, 8 h) NCI-H1299 cells. Cells were exposed to BTZ in FBS-free medium (Opti-MEM™); GM130 was used as a cell lysate-contamination control. Membranes were cut into fragments according to the proteins’ molecular weight. For chemiluminescent signal detection X-ray film was used. Original autoradiograms/immunoblots are presented in Fig. S5. (**b**) Number of particles isolated by SEC from conditioned cell culture media from non-treated (DMSO) or BTZ-treated cells, measured by nanoparticle tracking analysis (NTA). EVs numbers were calculated in relations to the number of cells. (**c**) Representative histogram of particle size distribution in EVs sample obtained from non-treated (DMSO) cells, calculated by NTA measurement.

In the next step we examined whether extracellular HSPA2 can be associated with EVs. Samples of purified EVs were isolated from conditioned media from NCI-H1299 cells using a well-established method combining differential centrifugation, ultrafiltration, and size exclusion chromatography (SEC)^43,44^. The single previously well-established and characterised^43^ SEC fraction enriched in approved markers (CD63, CD81, and TSG101) (Fig. 4a) and containing nanovesicle of expected sizes (mean size 105.6 + / −  0.6 nm, mode size 92.0 + / −  5.7 nm) was hereinafter referred as EVs sample. The Fig. 4c shows a representative nanoparticle tracking analysis **(**NTA)-based histogram of particles size distribution in EVs samples used in this study.

We found that SEC-purified EVs derived from BTZ-treated cells showed higher levels of HSPA2 and HSPA1 proteins, as well as CD63, CD9, CD81 EVs marker proteins as compared to non-treated cells (Fig. 4a). The GM130 protein (a negative EVs marker), had not been detected in EVs samples (Fig. 4a), thus the samples were free from contamination with cellular proteins. BTZ (32 nM) treatment nearly doubled the number of EVs as compared to drug-free conditions (Fig. 4b). These findings demonstrated that proteotoxic stress augments the extracellular release of HSPA2 and HSPA1 in association with EVs.

**Figure 5:**
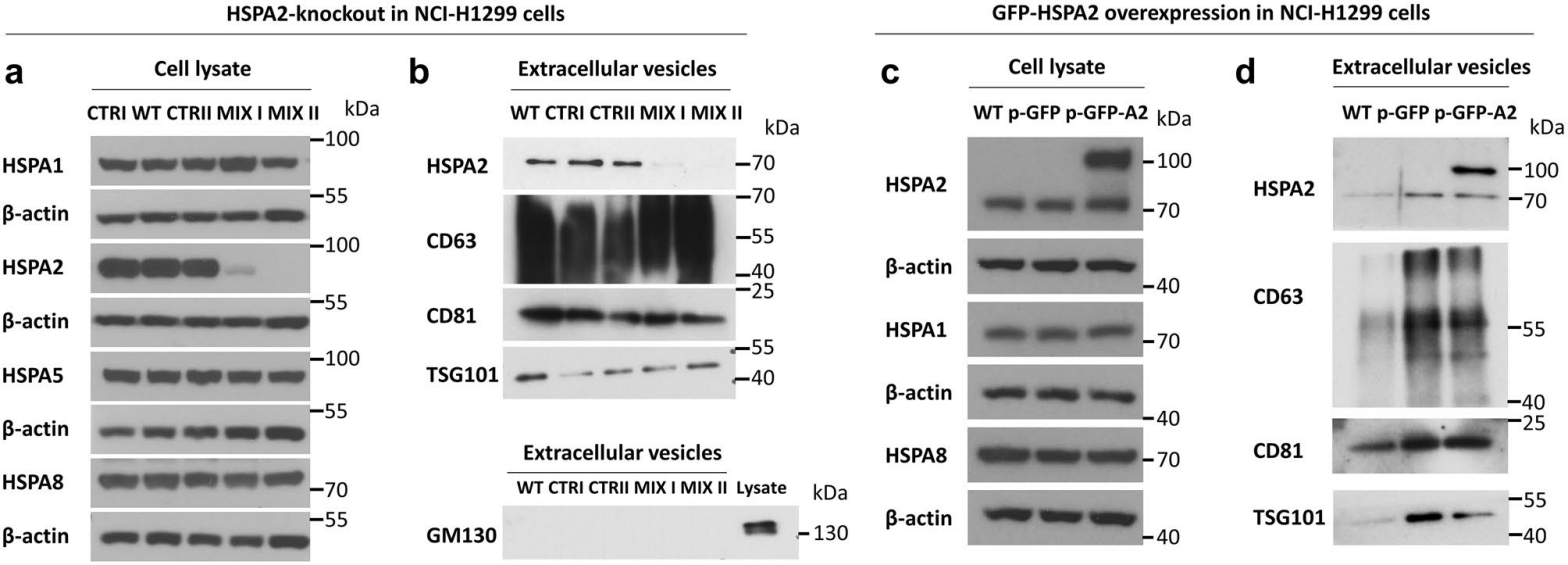
Specificity of HSPA2 detection in SEC-purified EVs derived from HSPA2-knockout and HSPA2-overproducing NCI-H1299 cells. (**a**) Analysis of HSPAs in HSPA2-knockout cells modified by CRISPR/Cas9 system. WT, wild-type cells, CTR I, CTR II, modified control cells; MIX I, MIX II; pools of isogenic clones with partial and full knockout of the *HSPA2* gene expression, respectively. (**b**) Levels of HSPA2; positive (CD63, CD81, TSG101) and negative (GM130) protein markers in EVs produced by wild-type (WT), modified control (CTR I, CTR II) and *HSPA2*-knockout cells (MIX I, MIX II). (**c**) Expression of HSPAs and GFP-HSPA2 fusion protein in WT, control GFP tag-overexpressing (p-GFP); GFP-HSPA2-overexpressing (p-GFP-A2) cells. Stable cell lines were established by lentiviral transduction. (**d**) Detection of HSPA2 in EVs-enriched SEC fraction derived from WT, control p-GFP and p-GFP-A2 cells. WT HSPA2, 70 kDa; GFP-HSPA2 fusion protein, 100 kDa. Experiments were repeated twice for each HSPA2 model (HSPA2-knockout or GFP-tagged). Representative immunoblots are presented (n = 2). β-actin was used as a protein loading control. Membranes were cut into two (or three) fragments according to the proteins’ molecular weight. For chemiluminescent signal detection X-ray film was used. Original autoradiograms/immunoblots are presented in Fig. S6.

As the members of the HSPA family share very high homology, they can be easily misidentified by non-specific immunodetection tools^34^. Therefore, in subsequent experiments we verified suitability of our anti-HSPA2 antibody for specific detection of EVs-loaded HSPA2. For this purpose, we used two stably modified pools (MIX I, MIX II) of HSPA2-knockout NCI-H1299 isogenic clones that were generated using CRISPR/Cas9 system (Fig. 5a). Cells of MIX I pool (6 clones) showed partial knockout of *HSPA2*, while of MIX II pool (8 clones) were HSPA2-null. The HSPA2-knockout cells, the control modified clones pools (CTRI, CTRII), and wild-type cells showed the same expression pattern of highly homologous HSPAs including HSPA1, HSPA8 and HSPA5 (Fig. 5a). Importantly, for the control, MIX I and MIX II pool cells the patterns of HSPA2 immunodetection were fully consistent in both SEC-purified EVs and the parental cells; HSPA2 was absent in EVs derived from HSPA2-null (MIX II) cells (Fig. 5b).

Additionally, we used stably modified NCI-H1299 cells overexpressing GFP-HSPA2 fusion protein (Fig. 5c). Analysis of cells overexpressing HSPA2 in fusion with GFP tag (p-GFP-A2 cells) and control cells overexpressing GFP only (p-GFP cells) revealed the same pattern of HSPA2-related bands in samples of the total cellular proteins (Fig. 5c) and the EVs-associated protein (Fig. 5d). In this model, EVs derived from control cells (p-GFP) contained only endogenous non-tagged HSPA2; while EVs from p-GFP-A2 cells contained GFP-tagged HSPA2 (Fig. 5d). It is worth noting that in our model overexpression of GFP-HSPA2 or GFP tag had no influence on expression of highly homologous HSPA1 and HSPA8 proteins in NCI-H1299 cells (Fig. 5c).

### HSPA2 is associated with SEC-purified EVs derived from human normal and cancer cell lines as well as human urine

**Figure 6:**
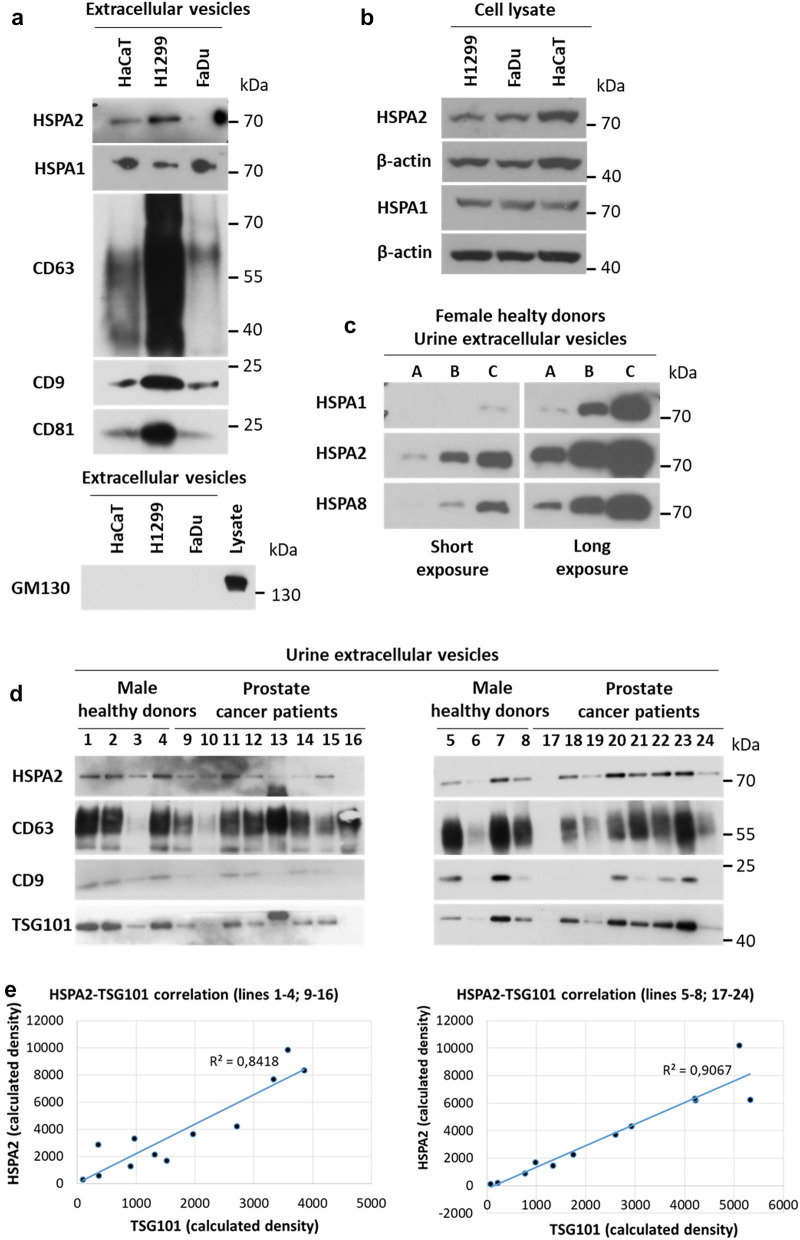
HSPA2 is present in small extracellular vesicles (EVs) derived from different biological sources. (**a**) Western blot detection of HSPA2 and HSPA1 in EVs released into cell culture media by non-cancerous (HaCaT) and cancer (NCI-H1299, FaDu) cells; CD63, CD9, CD81, the EVs protein markers; GM130, negative EVs marker. (**b**) Intracellular protein levels of HSPA2 and HSPA1. β-actin was used as a protein loading control. (**c**) Detection of HSPA1, HSPA2 and HSPA8 in SEC-purified EVs derived from urine of female healthy donors (A-C). (**d**) Detection of HSPA2 and EVs’ markers in SEC-purified EVs derived from urine of two independent groups of males, each compromising four healthy donors (samples 1–4; 5–8) and eight patients with prostate cancer (samples 9–16; 17–24). Membranes were cut into two (or more) fragments according to the proteins’ molecular weight. For chemiluminescent signal detection X-ray film was used. Original autoradiograms/immunoblots are presented in Fig. S7. (**e**) HSPA2-TSG101 densitometry correlation analysis; results obtained using ImageJ software ^46^;. Each dot represents intensity values of HSPA2 and TSG101 bands calculated from immunoblots shown in (**d**).

Subsequently, we searched HSPA2 in SEC-purified EVs derived from other types of biological samples. In addition to NCI-H1299 cells, epithelial keratinocyte HaCaT cell line and hypopharyngeal cancer FaDu cell line were selected for analysis. These cell lines showed similar intracellular levels of HSPA2 and HSPA1 proteins (Fig. 6b). Both proteins were detected in EVs derived from each of non-stressed cell lines (Fig. 6a). Interestingly, the level of HSPA2 (but not HSPA1) was corresponding to the levels of EVs protein markers (CD63, CD9, CD81; Fig. 6a). Negative results of the GM130 marker protein detection (Fig. 6a) confirmed a lack of contamination with cellular proteins in EVs-enriched samples.

Human urine, an easily accessible EVs-rich biofluid is suitable for isolation of high purity EVs samples due to very low level of background proteins^45^. We found that EVs derived from urine of three healthy female donors contained HSPA2, and also HSPA1 and HSPA8 proteins, albeit the protein levels varied between samples (Fig. 6c). HSPA2 was also found in EVs derived from urine of healthy male donors (n = 8) and prostate cancer patients with different Gleason Scores (n = 16) (Fig. 6d). WB analysis revealed a donor-to-donor variability in amounts of EVs-related proteins, and the levels of HSPA2 and TSG101 EVs markers were correlated (Fig. 6d,e).

### HSPA2 is present in human urine-derived EVs isolated by immunocapture method

**Figure 7:**
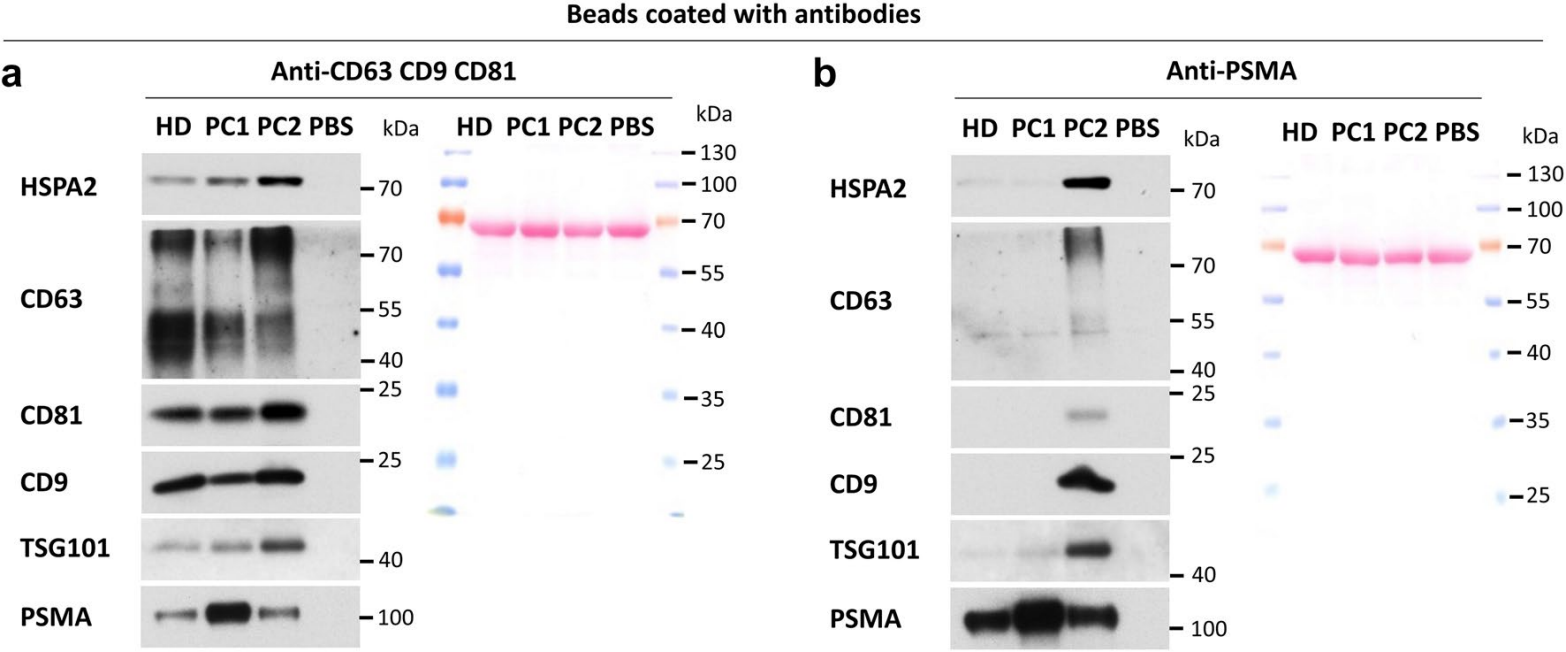
Immunodetection of HSPA2, EVs markers, and prostate-related marker (PSMA) in urinary EVs isolated using antibody-conjugated beads. (**a**) Beads were coated with the mixture of anti-CD63, anti-CD81 and anti-CD9 antibodies in equal proportions or (**b**) with anti-PSMA antibody. Urine samples from one male healthy donor (HD) and two prostate cancer patients (PC1; PC2) were tested. A sample with PBS and coated beads was used as a control for non-specific antibody interaction during Western blot (WB) procedure. Antibody fragments detached from the coated beads during samples preparation for WB were visualized with Ponceau S staining as a control of the sample loading and electrotransfer performance. Membranes were cut into fragments according to the proteins’ molecular weight. For chemiluminescent signal detection X-ray film was used. Original autoradiograms/immunoblots are presented in Fig. S8.

At this point we collected considerable amount of plausible evidence that HSPA2 is closely related to EVs, however we still needed direct evidence that not only is the protein present in the EVs sample but it is indeed a part of EVs’ cargo. To obtain a proof we isolated EVs using magnetic beads coated with the mixture of antibodies recognizing EVs-related proteins CD63, CD9, CD81. These beads allowed us to immunocapture EVs from EVs-enriched SEC fraction derived from the urine of one healthy donor and two prostate cancer patients. Also in this case we were able to easily immunodetect HSPA2 in urine-derived EVs (Fig. 7a). Moreover, the level of HSPA2 corresponded to the ones of EVs-related markers such as CD63, CD81, CD9 and TSG101 (Fig. 7a). Finally, we performed an additional proof-of-concept experiment aimed at targeting the prostate specific membrane antigen (PSMA) to immunocapture prostate tissue-related urinary EVs. We used beads coated with anti-PSMA antibodies to capture a fraction of urinary PSMA-positive EVs according to protocol described by Mizutani et al.^47^ that enabled successful immunocapture of EVs from plasma of prostate cancer patient. For isolation of PSMA-positive EVs we used the same urine samples as in the previous immunocapture experiment. We detected HSPA2 along with PSMA marker albeit in this case the levels of HSPA2 and EVs markers significantly varied across the samples (Fig. 7b). To rule out possible non-specific reaction between antibodies eluted from beads and antibodies exploited for immunodetection, control probe where PBS was added to coated beads instead of EVs was processed along with the test samples. Altogether, we proved that HSPA2 can be detected along with the whole set of markers of EVs even if the immunocapture was targeted at PSMA marker (Fig. 7b).

## Discussion

In the present study we have demonstrated that HSPA2, one of the most peculiar and least characterised members of the HSPA family, is released to extracellular space from both normal and cancer cells under physiological and stressful conditions. To the best of our knowledge, we are the first to identify HSPA2 as a basic component of the EVs cargo. We found that during proteasome inhibition, loading of HSPA2 into EVs is executed at the expense of lowering the intracellular levels thereof (Fig. 3). The same conditions also lead to increased release of HSPA1 in EVs, but in this case a preceding massive stress-related upregulation of the intracellular protein levels occurred. This may suggest that active targeting mechanisms may participate in HSPA2 loading into EVs upon proteotoxic stress. Bearing in mind that EVs are involved in response and/or adaptation to various cellular stresses that lead to proteostasis disturbance^15^, one may speculate that an increased release of HSPA1 and HSPA2 in EVs takes part in the proteostasis restoration mechanism.

The role of EVs in the proteostasis control appears to be complex. The cells can use EVs to dispose of oligomerised/aggregated or misfolded proteins, or to repair proteotoxic stress-induced protein damage in recipient cells^48,49^. In this aspect, EVs-mediated secretion and intercellular transmission of HSPs were shown to be responsible for maintenance of proteostasis at the organismal level^50^. In *Drosophila* fly such an EVs-dependent transport of HSPs may functionally compensate for the imbalances in the ability of different cells and tissues to activate cytoprotective stress response under stressful conditions^50^. Therefore, our results suggest that increased loading of HSPA2 and HSPA1 into EVs may be a part of non-cell-autonomous mechanisms of proteostasis control upon proteotoxic stress. In this context, our previously published results^33,35^ allow to speculate that HSPA paralogs could act in concert due to their high functional redundancy.

We have been able to easily detect HSPA2 in EVs derived from different biological sources. It is well known that EVs are released by all types of cells^9^ and their cargo consists of the set of characteristic proteins (including CD63, CD9, CD81, TSG101, or Alix) and certain other proteins specific for their parental cells^10,11^. This study also revealed that the levels of HSPA2 in urinary EVs from healthy donors as well as prostate cancer patients were correlated with EVs markers, especially TSG101. Therefore, we came to the conclusion that not only is HSPA2 a component of the basic set of the cargo of the small EVs, but it also can be considered EVs marker, at least for urinal EVs. Further research performed using other human biofluids should be undertaken to examine whether HSPA2 can be regarded as a universal EVs marker.

Heat shock proteins such as HSPA (HSP70) and HSPC2 (HSP90AB1) are commonly considered as the constitutive EV proteins^20,21^, are listed among the EVs markers^51^. HSPA-EVs have been also reported as potential cancer^52^ or neurodegenerative disease^53^ biomarkers. Unfortunately, a large number of reports that aimed at characterising extracellular EVs only invoke the name HSP70 (HSPA) without a precise indication which member of HSP70/HSPA family is actually considered. Such a referencing does not meet the guidelines for the nomenclature of the human HSP proteins^54^, according to which, the term HSP70/HSPA refers to the whole HSPA family. In this study, we were able to detect three different members of HSPA family, namely HSPA2, HSPA1, and HSPA8 as EVs cargo using verified HSPA paralog-specific antibodies^34^.

The majority of urinary EVs originate from cells lining the nephron lumen and the urinary tract, as well as from infiltrating inflammatory cells^55^, whereas, urine also contains the fraction of prostate-derived EVs. The hypothetical mechanism that would explain the presence of urinal prostate-derived EVs is related to draining of prostatic fluid during urination in normal conditions^56,57^. It is believed that urine, at least in physiological conditions, is devoid of plasma EVs as vesicles cannot pass through the glomerular filtration machinery. However, this view is challenged by the results of studies aimed at identifying EVs-related biomarkers for non-invasive diagnostic of lung or breast cancer in urine^58,59^. Thus, it appears that the origin of the urinary EVs repertoire is mixed and is yet to be fully uncovered.

HSPA2 is present in the testis and several somatic organs, showing cell-type dependent expression pattern^26^. In the kidney cortex, HSPA2 was detected in epithelial cells of the distal tubules, but was not in other types of tubules, glomeruli, or Bowman’s capsule, neither was it in the bladder and prostate epithelia^26^. Since EVs mirror the cell of origin, we hypothesize that epithelial cells lining the kidney distal tubules are a source of HSPA2-loaded urinal EVs, at least in physiological conditions. On the basis of our previous study, it can be anticipated that HSPA2-positive prostate cancer cells^32^ are the potential origin of HSPA2-loaded PSMA-positive EVs. HSPA2 is also abundant in multilayered/pseudolayered epithelia covering the skin, esophagus, or bronchi^26^. Here, we demonstrated that keratinocytes, a major cell type in epidermis, release HSPA2-loaded EVs in vitro*,* too. Proteomic data provided by Klingeborn et al.^60^ revealed the presence of HSPA2 among the proteins enriched in EVs released by retinal pigmented epithelium. Altogether, these data shed a new light on HSPA2 as an extracellular chaperone. Given that EVs, as indicated by the presence of well-approved EVs marker proteins, were released from *HSPA2-null* cells, a potential role of HSPA2 for EVs biogenesis can be excluded. However, it would be desirable to examine whether HSPA2-loaded and HSPA2-absent EVs could deliver different functional messages to recipient cells.

At present, the discussion on the potential biological significance of extracellular HSPA2 can be speculative. It is well known that non-cytosolic HSPAs can be located onto plasma membrane, or can be released from cells in a soluble or EVs-bound form^22,61^. The transfer of HSPA outside the cells is stimulated by stress challenges, including pathophysiological states such as cancer or neurodegenerative disorders. Extracellular HSPA represents a signal that can be recognised by the immune system and has immunomodulatory importance (review in^62^). Moreover, exosomal HSPA appears to have dual immunostimulatory or immunosuppressive roles. For example, tumour-specific membrane-bound exosomal HSPA can trigger an anti-tumour response via stimulation of cytotoxic and NK lymphocytes^63,64^. In contrast HSPA-loaded EVs were also shown to provoke enhanced tumourigenicity via stimulating immunospressive activity of myeloid-derived suppressor cells^65^. Therefore, it is important to investigate the immunomodulatory properties of HSPA-loaded EVs, among other by addressing the questions of what is the role of HSPA2 in EVs released from non-cancerous cells at physiological conditions and whether it has immunomodulatory properties. Taking into account that several HSPA paralogs can be loaded into EVs, it is not known whether a potential impact of a particular HSPA can be specific or redundant with other HSPAs. It is noteworthy that in spite of high homology between HSPA paralogs, each of them admits unique immunomodulatory epitopes, or can carry a different set (at least partially) of antigenic peptides. Consequently, the question of redundancy or, at least, partial paralog idiosyncrasy of HSPA family members in the immunomodulatory context arises naturally. The above questions should be addressed while taking into account that HSPA paralogs are expressed differently as per cell type and exhibit different induction levels under environmental and/or pathophysiological stress conditions.

During our work on EVs we gained awareness of a potential risk of creating artifacts. One of the major challenges in studies on EVs cargo are contaminants co-isolated with extracellular vesicles that can lead to mis- or over-interpretation of the results^66^. In the in vitro studies the main source of “contaminants” is fetal bovine serum (FBS), which delivers both bovine EVs as well as significant amount of serum proteins that are difficult to remove when isolating EVs. Therefore, to ensure both quality and purity of EVs samples we used EVs preparation methods living up to exacting standards of proteomic studies, namely size exclusion chromatography combined with various centrifugation and ultrafiltration techniques^66^. Moreover, in order to warrant the purity of the sample and increase the results credibility, commercial EVs-depleted FBS with reduced levels of serum proteins was used^43^, whereas short-term experiments were performed using cell culture medium without FBS supplementation. In the experiments with EVs derived from biofluids in order to reduce the risk of co-isolation contamination, we used urine which, compared to blood plasma, has a considerably lower amount of background particles (such as lipoproteins and proteins). Additionally, in order to meet the requirements of the International Society for Extracellular Vesicles we confirmed the presence of vesicles of the appropriate size using the NTA method^21^.

Furthermore, employment of the immunoenrichment method for EVs isolation allowed us to confirm that HSPA2 is a component of EVs cargo. At this point, we proved that after immunocapture with magnetic beads coated with antibodies capable of recognising various characteristic EVs proteins, a strong signal of HSPA2 remained detectable along EVs markers used for immunocapture, and also one independent marker not targeted during isolation (TSG101). Finally, HSPA2 along with all the above-mentioned EVs markers, were detected in PSMA-positive urinal EVs. PSMA was previously reported as an efficient target for isolation of prostate-derived EVs from plasma^47^ and urine^67^. And indeed, HSPA2 was detected along with PSMA and EVs markers, albeit in this case enrichment levels of the investigated proteins differed across the samples as could have been expected (Fig. 7b). Padda et al.^68^ showed that the majority of plasma vesicles released by prostate cells (PSMA +), investigated by flow cytometry, do not co-express markers such as CD63, CD81, or CD9. Also Mizutani et al.^47^ observed the difference in the CD9 protein enrichment levels in PSMA-positive EVs. Since data presented in Fig. 7a concern a mixed EVs population, the results may differ from the ones obtained for a single subpopulation of PSMA-positive EVs (Fig. 7b). Regardless of these considerations, we have proved that even in the case of different immunocapture target proteins, HSPA2 accompanies proteins characteristic for small EVs.

Taking the above findings into account, we confirmed that HSPA2 loading into EVs occurred in normal and cancer cells under physiological and proteotoxic stress conditions, however its localisation in EVs (membrane or internal) requires further investigation. Our findings justify subsequent studies on HSPA2 as a EVs cargo, potentially involved in cell-to-cell signaling.

## Materials and methods

### Cell culture and experimental conditions

NCI-H1299 (non-small cell lung carcinoma, CRL-5803), MCF7 (breast carcinoma, HTB-22), and FaDu cells (squamous cell carcinoma, pharynx, HTB-43) were purchased from ATCC (Manassas, VA, USA). HaCaT (spontaneously immortalized keratinocytes) cell line was acquired from CSL Cell Line Service GmbH (Eppelheim, Germany). Cells were cultured at 37 °C under standard conditions (5% CO_2_, 95% humidity, 21% O_2_ concentration). NCI-H1299, MCF7 and FaDu cell lines were grown in RPMI, DMEM-F12 or MEM (Sigma-Aldrich, Merck KGaA, Darmstadt, Germany) respectively. HaCaT cells were grown in DMEM (high glucose 4.5 g/L; Sigma-Aldrich, Merck KGaA, Darmstadt, Germany). Cell culture media were supplemented with 10% heat-inactivated fetal bovine serum (EuRx, Gdańsk, Poland) and antibiotics (gentamycin or penicillin-streptomycin). Cells were regularly checked for mycoplasma contamination.

### Incubation experiments

The following stock solutions were used in the incubation experiments: manumycin A (MA; 10 mM in DMSO; Sigma-Aldrich, Merck KGaA, Darmstadt, Germany), MG132 (10 mM in DMSO; Selleck Chemicals, Houston, TX, USA), VER-155008 (VER; 20 mM in DMSO; Merck KGaA, Darmstadt, Germany), bortezomib (BTZ; 1.6 mM in DMSO; Selleckchem, Houston, TX, USA), bafilomycin A1 (BAF; 0.1 mM in DMSO; Sigma-Aldrich, Merck KGaA, Darmstadt, Germany), chloroquine (ChQ; 50 mM in DMSO; Sigma-Aldrich, Merck KGaA, Darmstadt, Germany). Working solutions were freshly prepared from stock solutions prior to each experiment (in a culture medium or Optimem (Opti-MEM™ Reduced Serum Medium, GlutaMAX™ Supplement; Gibco, Thermo Fisher Scientific, Waltham, MA, USA)). Control cells were incubated with medium containing dimethyl sulfoxide (DMSO). All experiments were performed without the antibiotics addition.

### LDH release assay

Cells (NCI-H1299) were plated into 10 cm dishes, cell culture medium was changed after 24 h culture to Opti-MEM™ Reduced Serum Medium (without phenol red, with GlutaMAX supplement; Gibco Thermo Fisher Scientific, Waltham, MA, USA) with or without BTZ for the indicated time. The cytotoxic effect was measured using CytoTox 96 Non-Radioactive Cytotoxicity Assay according to the manufacturer’s protocol (Promega; Madison, WI, USA). The absorbance at 490 nm was measured using a microplate reader.

### RNA isolation and RT‐qPCR analysis

The day after seeding, the cells (at a maximum confluence of 50–60%) were exposed to a drug*-*containing medium for indicated time. Cells were harvested and total RNA was isolated using Nucleospin RNA Plus kit (Macherey‐Nagel, Germany) in accordance with manufacturer’s protocol. cDNA synthesis and RT‐qPCR reactions were performed as previously described^28^. Relative gene expression was calculated using the 2^(‐ΔΔCt)^ method and normalised to the reference genes (RPL13, B2M, TMEM43) expression. Reference genes were selected individually for each cell line and treatment condition (Table S1). Sequences of starters read as follows: HSPA2, forward (F) 5′-TTGCAACCCCATCATCAGCA-3′, reverse (R) 5′-TTGGCACAAGGACATTTCAAAGA-3′; HSPA1A, F 5’-AGCTGGAGCAGGTGTGTAACCC-3’, R 5’-AAAAACAGCAATCTTGGAAAGGCCC-3’; RPL13A, F 5’-CCCTACGACAAGAAAAAGCGG-3’, R 5’-TCCGGTAGTGGATCTTGGCT-3’; B2M, F 5ʹ-CTGGGTTTCATCCATCCGACA-3ʹ; R 5ʹ-GTCTCGATCCCACTTAACTATCTTGG-3ʹ; TMEM43, F 5’-CTTGTGGTGTCTCCCGACAG-3’; R 5’-TTGGTACATCTCCACGTGCC-3’.

### Generation of modified cell lines

The CRISPR/Cas9-modified cell lines were generated as described previously^29^. Briefly, NCI-H1299 cells were transfected with HSPA2 Double Nickase Plasmid (sc-400832-NIC, Santa Cruz Biotechnology, Inc., Dallas, TX, USA) and Control Double Nickase Plasmid (sc-437281, Santa Cruz Biotechnology). Subsequently, GFP-positive control cells and *HSPA2*-knockout cells were sorted using the BD FACS Aria III Cell Sorter (BD Bioscience, San Jose, CA, USA) and plated onto 6 cm plates. Afterwards, single clones were obtained by limiting dilution on 96-well plates. Disruption of the *HSPA2* gene expression in clones was examined by WB. The HSPA2 partial-knockout clones were pooled and denoted MIX I; HSPA2 full-knockout clones were pooled and named MIX II. HSPA2-overexpressing cell lines were generated using lentiviral gene transfer system as described previously^34^. Briefly, the pLVX-Puro-GFP-HSPA2 plasmid encoding the GFP-HSPA2 fusion protein and the control pLVX-Puro-GFP plasmid encoding GFP tag only were constructed by insertion of the corresponding coding nucleotide sequences into EcoRI and BamHI restriction sites of the pLVX-Puro lentiviral vector (Clontech, Takara Bio, Mountain View, CA, USA). For DNA cloning In-Fusion® HD EcoDry™ Cloning Plus (Clontech, Takara Bio, Mountain View, CA, USA) kit was used according to the manufacturer’s manual. *GFP* sequence was cloned from pEGFP-C2 plasmid (Addgene, Watertown, MA, USA). Primers used in cloning were as follows: F 5’- CTCAAGCTTCGAATTCATGGTGAGCAAGGGCGAGG 3’ (common for both plasmids cloning), R 5’ TAGAGTCGCGGGATCCTTACTTGTACAGCTCGTCCATGCC 3’ (for GFP cloning), R 5’ TAGAGTCGCGGGATCTTAATCCACTTCTTCGATGGTGGG 3’ (for GFP-HSPA2 cloning). The nucleotide sequence coding for GFP-HSPA2 fusion protein was generated by subcloning the HSPA2 coding sequence from pEF1-HSPA2 plasmid^31^ into EcoRI and BamHI restriction sites of pEGFP-C2 plasmid. Details on cloning procedures are available on request. The correctness of plasmids generation was confirmed by Sanger sequencing. The pLVX-Puro-GFP-HSPA2 and pLVX-Puro-GFP plasmids served to produce lentiviruses using Lenti-X shRNA Expression System (Clontech, Takara Bio, Mountain View, CA, USA). Conditioned lentivirus-containing media were used for transduction of NCI-H1299 cells as described previously^33,34^. Stably transduced cells were selected using puromycin (1 μg/ml, Sigma Aldrich, St. Louis, MO, USA).

### Analysis of conditioned medium

NCI-H1299 cells were plated onto 10 cm dishes. Prior to the experiment the cells were washed three times with PBS to remove the residual albumins and other FBS-related proteins. The cells were incubated in a drug solution (in Opti-MEM™) for 8 h or 24 h. Then, the conditioned medium was collected and cleared by centrifugation at 200, 2000 and 10,000 × g for 2 × 10 and 30 min, respectively. Subsequently, medium was concentrated up to 100 µL with Vivaspin 20 100,000 MWCO (Sartorius, Goettingen, Germany). The protein concentration was measured using Protein Assay Kit (Bio-Rad; Hercules, CA, USA); levels of selected proteins were analysed by WB.

### Urine collection

Human urine samples were obtained from healthy donors (3 females and 8 males) as well as from male patients (n = 16) with locally advanced prostate cancer (Gleason score of 7 or 9) who had been undergoing hormone therapy. All study participants were Caucasians; females at the age range from 31 to 55 years, males at the age range from 55 to 77 years. Samples were collected during the first-in-day urination. Directly after delivery, samples were transferred to sterile tubes and centrifuged for 5 min at 1000 rpm at 4 °C. The collected supernatant was preserved in fresh sterile tubes at − 80 °C for further investigation. The study was approved by the local Bioethics Committee (approval no. KB/430-57/18). All urine donors provided Informed Consent indicating their conscious and voluntary participation. The study was performed in accordance with the Declaration of Helsinki and relevant guidelines/regulations.

### EVs isolation

NCI-H1299, HaCaT and FaDu cells were cultured in T-175 flasks (Nunc, Thermo Fisher Scientific, Waltham, MA). Standard culture medium was replaced with culture medium containing 5% (v/v) exosome-depleted FBS (Thermo Fisher Scientific, Waltham, MA, USA) at 24 h prior to the experiment, unless indicated otherwise.

EVs were isolated using size exclusion chromatography as described previously^43,44^. The conditioned cell culture medium and human urine were cleared by centrifugation at 200, 2000, and 10,000 × g for 2 × 10 and 30 min, respectively. A fixed-angle rotor was used. The supernatant was filtered through sterile syringe filter with a 0.22 µm pore size hydrophilic polyethersulfone (PES) membrane (Sigma-Aldrich, Merck KGaA, Darmstadt, Germany) and concentrated up to 1 ml using Vivaspin 20 100,000 MWCO ultrafiltration devices (Sartorius, Goettingen, Germany). Subsequently 1 ml of concentrate was loaded onto the chromatography column (Bio-Rad; Hercules, CA, USA) filled with 10 ml of Sepharose CL 2B (GE Healthcare, GE17-0140-01). Fractions (1 ml) were eluted with PBS without divalent cations. The first fraction was collected right after the sample had been loaded. The most enriched fraction (the same for all samples) was used for further studies as ‘EVs fraction’. Imaging of EVs in the urinal ‘EVs fraction’ was performed by transmission electron microscopy (Fig. S1).

### EVs immunocapture

Dynabeads superparamagnetic polystyrene beads (4.5 μm diameter) coated with streptavidin (Invitrogen, Thermo Fisher Scientific, Waltham, MA, USA) were used according to manufacturer instructions with some minor modifications. Briefly, 1.5 ml of magnetic beads suspension was transferred to a new tube and washed twice with PBS with 0.1% BSA. Then magnetic beads were resuspended in 1.5 ml of PBS and 0.1% BSA with 6 μg of antibody and incubated for 2 h at room temperature with slow orbital rotation. All types of beads (anti-CD63, anti-CD81, anti-CD9, and anti-PSMA) were processed separately. After triple wash of antibody-coupled beads with PBS with 0.1% BSA a third part of each sample containing anti-CD antibodies was mixed to obtain one sample with equal proportions of anti-CD63, anti-CD9, and anti-CD81 beads. Directly after the final wash anti-CD63/CD81/CD9 or anti-PSMA beads were distributed into four tubes and appropriate pre-enriched EVs sample (SEC fraction) or PBS were added (300 μl). Each sample contained the same protein level. The coupled beads were gently resuspended by shaking; no vortexing was used. Then the samples were incubated for 24 h at 4 °C with slow orbital mixing. Subsequently, the samples were briefly centrifuged and washed twice with 1 ml of PBS with 0.1% BSA with gentle shaking; no vortexing was used. After the final wash, the samples were distributed into new tubes and the EVs proteins were extracted by boiling for 5 min in WB loading buffer. After separation on the magnet, the samples were used for WB analysis.

### Nanoparticle tracking analysis (NTA)

Particle concentration and size distribution in EVs samples were analyzed using NanoSight NS300 analyzer (Malvern Pananalytical, Malvern, UK). EVs were diluted in sterile-filtered Dulbecco's phosphate buffered saline (DPBS) without Ca^2+^ and Mg^2+^ (Lonza, Basel, Switzerland) to reach particle concentration optimal for the measurement range of the instrument. Three 1-min tracking repetitions of each sample were collected using syringe pump flow mode, with camera level of 12. Data were acquired and processed by the NTA Software ver. 3.4 (Malvern Pananalytical), with the threshold parameter set on 2.

### Western blot (WB) analysis of cellular and EVs proteins

For preparation of total cellular protein extracts cells were plated onto 6 cm dishes with a maximum confluence of 50–60%. The cells at 24 h after plating were exposed to the drugs or left untreated for 8 or 24 h. The total protein extracts were prepared by scrapping the cells in RIPA buffer (1 × PBS, 1% NP-40, 0.1% SDS, 0.5% SDC, 50 mM NaF, 1 mM PMSF) supplemented with a protease inhibitor mixture (Roche Molecular Systems, Inc; Rotkreuz, Switzerland). The samples were incubated on ice for 15 min and lysates were centrifuged (4 °C for 15 min at 22,000 × g).

*For EVs protein extraction*, preceded by the measurement of protein concentration, the mixture of EVs was mixed with WB loading buffer (12% SDS, 0.6% bromophenol blue, 60% glycerol, and optionally 600 mM DTT) at the v/v ratio of 1:5 and incubated for 10 min at 95 °C.

*WB analysis* of cellular and EVs proteins was performed under conditions individually optimized for each type of input. For total cellular protein lysates, protein concentration was determined using Protein Assay Kit (Bio-Rad; Hercules, CA, USA) in accordance with manufacturer’s instructions. The appropriate amount of protein was fractionated by SDS-PAGE on 8 or 12% polyacrylamide gels and transferred onto nitrocellulose membrane using Trans Blot Turbo system (Bio-Rad; Hercules, CA, USA) for 10 min. The membranes were blocked (60 min) in 5% nonfat milk/TTBS (0.25 M Tris–HCl (pH 7.5), 0.15 M NaCl, and 0.1% Tween-20). Prior to hybridization with primary antibodies the blot was cut into two or more fragments relative to the position of the size marker with appropriate margins to ensure central location of the protein of interest on the membrane fragment. The membranes were incubated with primary antibodies overnight at 4 °C (Table S2). The antibody–antigen interaction was detected using secondary antibodies (Table S2) and visualised using Clarity ECL Western Blot Substrate (Bio-Rad; Hercules, CA, USA) or WesternBright Quantum HRP substrate (Advansta; San Jose, CA, USA). X-ray films (Carestream Health, Inc, Rochester, N, USA) were used for chemiluminescent signal detection. β-actin was used as a protein loading control.

In the case of analysis of EVs proteins the WB protocol was modified as follow: protein concentration was determined using microBCA Protein Assay Kit (Thermo Fisher Scientific, Waltham, MA, USA) in accordance with manufacturer’s instructions; gel-fractionated proteins were blotted using standard wet electrotransfer (100 V) for 60 min (Bio-Rad; Hercules, CA, USA); WesternBright Sirius HRP substrate (Advansta; San Jose, CA, USA) was used for visualization of immunoreaction; CD63 and CD81 Exosomal marker proteins were detected under non-reducing conditions.

### Statistical analysis

Information on number of experiment repeats is placed in figures captions. The results are presented as the mean ± SD. The statistical analysis was performed by the two-tailed t-test using Microsoft Excel software. All significant results are shown in figures as appropriate where *p < 0.05; **p < 0.01; ***p < 0.001. R^2^ value was calculated using Microsoft Excel software.

## Supplementary Information

Supplementary Information.

## Data Availability

All materials and data are available upon reasonable request to the corresponding author.
