# Supplementary material for: Heat shock protein A2 is a novel extracellular vesicle-associated protein
Source: Sci Rep. 2023 Mar 23;13:4734. doi: 10.1038/s41598-023-31962-5 (PMC10036471; doi:10.1038/s41598-023-31962-5)
Supplement: Supplementary file 1 — Supplementary Information. [file 41598_2023_31962_MOESM1_ESM.pdf]

Supplementary Information

Heat Shock Protein A2 is a novel extracellular vesicle-associated protein

Damian Robert Sojka[1*], Agata Abramowicz[1*], Małgorzata Adamiec-Organiściok[1,2], Elżbieta Karnas[3], Łukasz Mielańczyk[4], Daria Kania[1], Sławomir Blamek[1], Ewa Telka[1], Dorota Scieglinska[1#]

[1] Maria Sklodowska-Curie National Research Institute of Oncology, Gliwice Branch, Wybrzeze Armii Krajowej 15, 44-102 Gliwice, Poland

[2] Department of Systems Biology and Engineering, Silesian University of Technology, Institute of Automatic Control, Akademicka 16, 44-100 Gliwice, Poland

[3] Faculty of Biochemistry, Biophysics and Biotechnology, Jagiellonian University, Gronostajowa 7 St., 30-387 Krakow, Poland

[4] Department of Histology and Cell Pathology, Faculty of Medical Sciences in Zabrze, Medical University of Silesia, Katowice, Poland

* These authors contributed equally

Corresponding author

[#]Dorota Scieglinska, dorota.scieglinska@io.gliwice.pl, tel: (48) 32 27 89 679, fax: (48) 32 231 35 12

Table S1. List of reference genes used in qPCR analysis.

Cell Line	Treatment	Analyzed gene	Reference genes
**NCI-H1299**	MA	HSPA2	*B2M*, *RPL13*
	MA	HSPA1	*B2M*, *RPL13*,*TMEM43*
	MG132	HSPA2	*RPL13*,*TMEM43*
	MG132	HSPA1	*RPL13*,*TMEM43*
	BTZ	HSPA2	*B2M*
	BTZ	HSPA1	*RPL13*,*TMEM43*
**NCI-H23**	MA	HSPA2	*RPL13*
	BTZ	HSPA2	*RPL13*,*TMEM43*
**MCF7**	MG132	HSPA2	*TMPR1*, *B2M*

Abbreviations: BTZ, bortezomib; MA, manumycin A; *RPL13*, Ribosomal Protein L13; *B2M*, Beta-2-Microglobulin; *TMEM43*, Transmembrane Protein 43.

Table S2. List of antibodies used in Western blot and EVs immunocapture analyses.

	Host/Clonality	Clone	Catalog Number/ RRID	Source	Dilution
**Western blot**					
**HSPA1**	Mo/M	C92F3A-5	ADI-SPA-810-F/AB_311860	Enzo, Life Sciences, Famingdale, NY, USA	1:5000 or 1:3000 for EVs samples
**HSPA2**	Rb/M	EPR4596	Ab108416/AB_10862351	Abcam, Cambridge, UK	1:5000 or 1:3000 for EVs samples
**HSPA5**	Mo/M	A-10	Sc-376768/nd	Santa Cruz Biotechnology, Inc., Dallas, USA	1:1000
**HSPA8**	Mo/M	B-6	Sc-7298/AB_627761	Santa Cruz Biotechnology, Inc., Dallas, USA	1:7500 or 1:3000 for EVs samples
**HSPC**	Mo/M	AC88	ADI-SPA-830-F/AB_11181197	Enzo, Life Sciences, Famingdale, NY, USA	1:2000
**p62**	Mo/M	D-3	Sc-28359/AB_628279	Santa Cruz Biotechnology, Inc., Dallas, USA	1:2000
**β-actin (HRP)**	Mo/M	AC15	A3854/AB_262011	Merck KGaA, Darmstadt, Germany	1:20000
**CD63**	Mo/M	MX-49.129.5	sc-5275/ AB_627877	Santa Cruz Biotechnology, Inc., Dallas, USA	1:1500
**CD63**[a]	Rb/M	SY21-02	MA5-32085/ AB_2809379	Thermo Fisher Scientific, Waltham, USA	1:1000
**CD81**	Mo/M	1.3.3.22	orb388959	Biorbyt Ltd.5, Cambridge, United Kingdom	1:500
**CD81**[a]	Rb/M	D3N2D	56039	Cell Signaling Technology, Danvers, USA	1:800
**CD9**	Mo/M	C-4	sc-13118/ AB_627213	Santa Cruz Biotechnology, Inc., Dallas, USA	1:800

**CD9**[a]	Rb/M	D8O1A	3174/ AB_2798139	Cell Signaling Technology, Danvers, USA	1:1000
**TSG101**	Mo/M	51	612697/ AB_399937	BD Biosciences, Franklin Lakes, USA	1:1000
**TSG101**	Rb/M	JJ0900	MA5-32463/ AB_2809740	Thermo Fisher Scientific, Waltham, USA	1:1000
**PSMA**	Rb/M	D4S1F	12702/ AB_2797998	Cell Signaling Technology, Danvers, USA	1:1000
**GM-130**	Rb/M	D6B1	12480/ AB_2797933	Cell Signaling Technology, Danvers, USA	1:1000
**Secondary**					
**Anti-Mo IgG (HRP)**	Go		AP124P/AB	Millipore, Billerica, MA, USA	1:5000
**Anti-Ra IgG (HRP)**	Go		AP132P/AB	Millipore, Billerica, MA, USA	1:2000
**Anti-Mo IgG (HRP)**	Go		31430/AB_228307	Thermo Fisher Scientific, Waltham, USA	1:10000
**Anti-Ra IgG (HRP)**	Go		111-035-003/ AB_2313567	Jackson ImmunoResearch Laboratories Inc., Ely, Cambridgeshire,, UK	1:10000

EVs immunocapture:

	**Host/Clonality**	**Clone**	**Catalog Number/ RRID**	**Source**	**Conjugate**
**CD63**	Mo/M	C047-1	MEX002-6/ nd	Medical & Biological Laboratories (MBL), Nagoya, Japan	Biotin
**CD9**	Mo/M	A100-4	MEX001-6/ nd	Medical & Biological Laboratories (MBL), Nagoya, Japan	Biotin
**CD81**	Mo/M	A103-10	MEX003-6/ nd	Medical & Biological Laboratories (MBL), Nagoya, Japan	Biotin
**PSMA (FOLH1)**	Mo/M	LNI-17	342510/ AB_2563243	BioLegend, San Diego, USA	Biotin

Abbreviations: RRID, Research Resource Identifier; EVs, Extracellular vesicles; M, monoclonal; Go, goat; Mo, mouse; nd, no data; Rb, Rabbit; HRP, horseradish peroxidase, [a] – antibodies used in Western Blot analysis of proteins in EVs isolated by immunocapture method

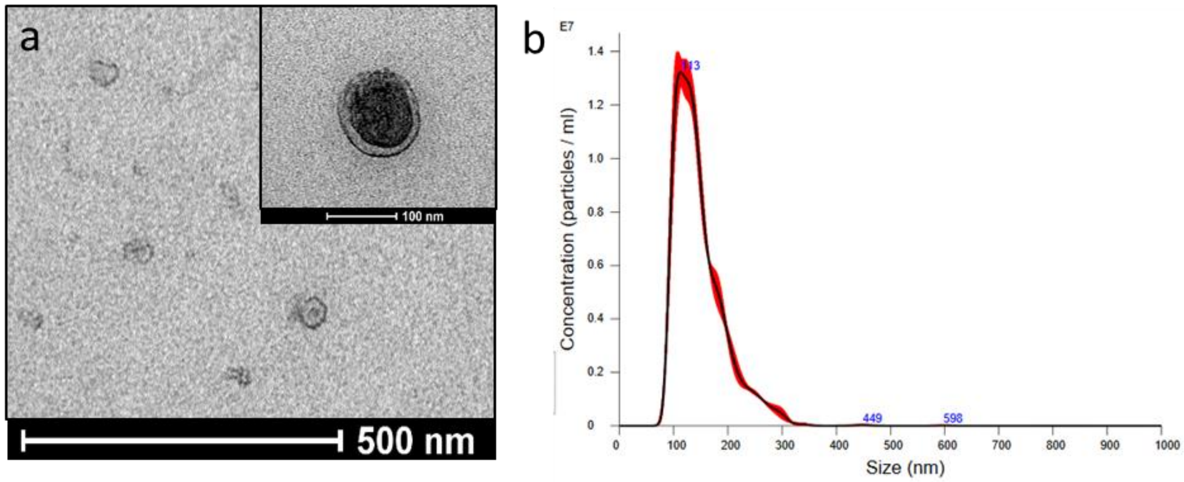

Figure S1. Characteristic of urinary EVs isolated by size exclusion chromatography. **(a)** Representative images from transmission electron microscopy. Two images with different magnifications are shown. Scale bar has been marked under the picture. **(b)** Representative histogram of particle size distribution of urinary EVs, mode: 112,3 nm.

Directly after size exclusion chromatography suspension containing urinal EVs fraction was mixed with equal volume of 4% PFA and stored at 4ºC until analysis in transmission electron microscope (TEM). For TEM analysis 5µl of the mixture was deposited onto a carbon-coated EM grid and incubated for 20 min in a dry environment. Subsequently, the grid has been washed for 2 min in PBS droplet placed on a sheet of Parafilm (Bemis Co. Inc., Neenah, WI). Afterwards, grid was successively transferred to the droplets of 1% glutaraldehyde (5 min,) and distilled water (2 min, 7 times), uranyl-oxalate solution (pH 7; 5 min) and methyl-cellulose-UA (10 min) prepared by mixing a 4% uranyl acetate solution and a 2% methyl-cellulose solution at the ratio of 1:9 (v/v). Finally the air-dried grids were visualized using a Tecnai G2 Spirit BioTWIN (FEI Companye, Hillsboro, OR) at 120-kV acceleration.

Fig. 1A

MG132

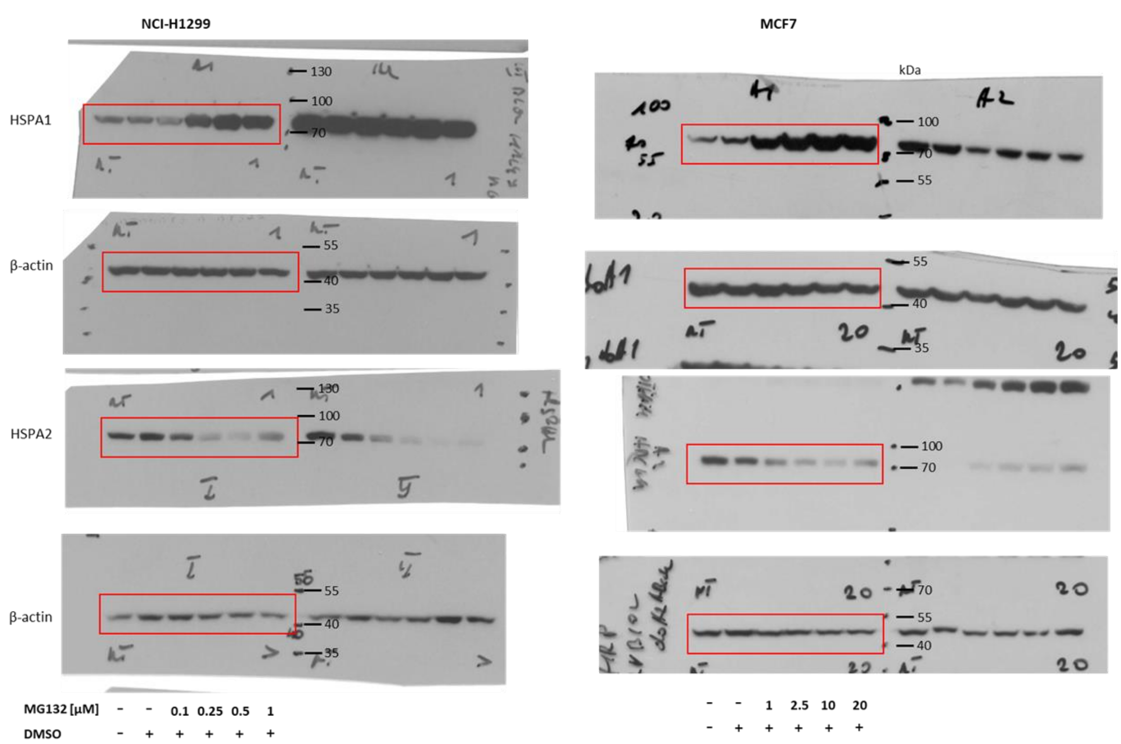

Fig. 1B

Bortezomib

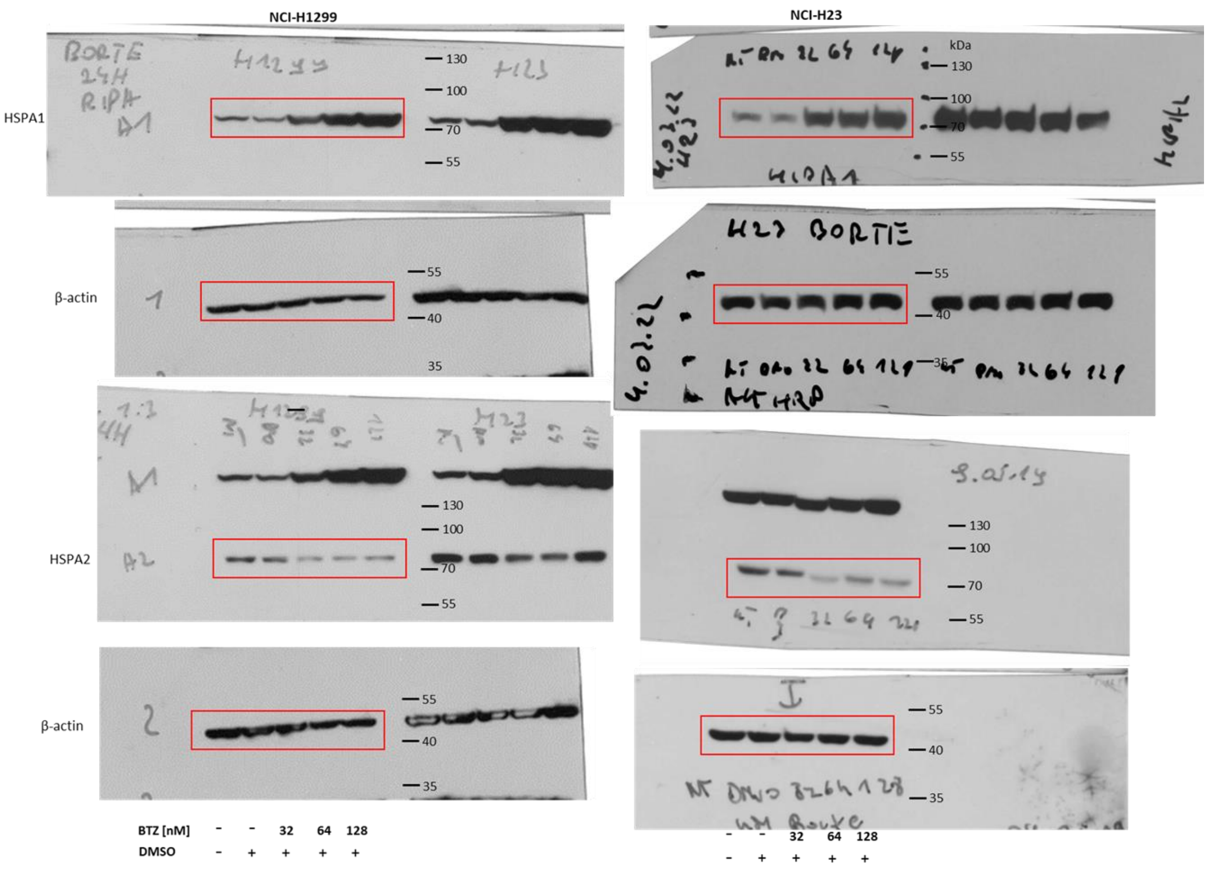

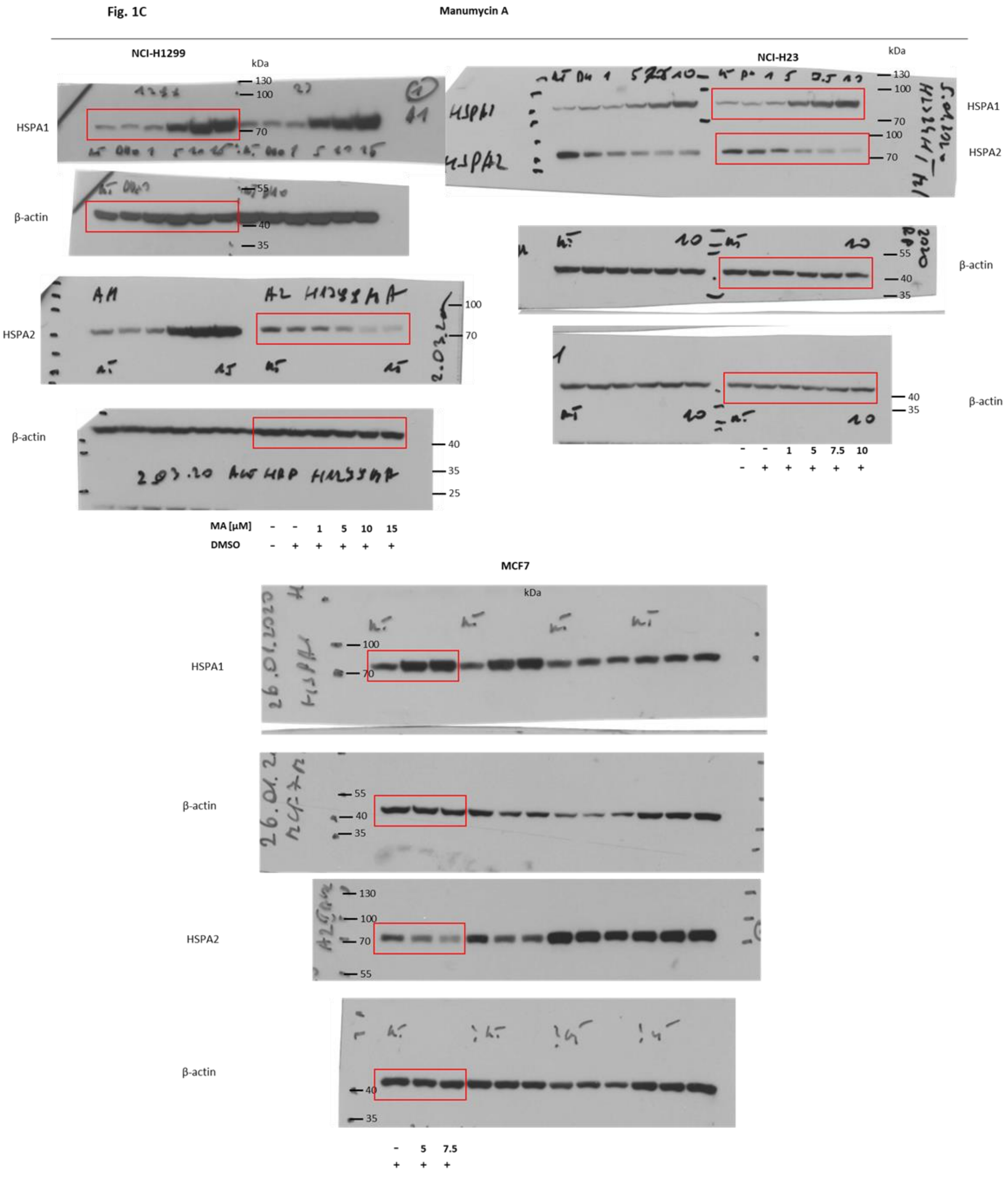

Figure S2. Unprocessed original scans of autoradiograms (immunoblots) included in Fig. 1 A-C. After protein transfer, membranes were cut according to 55 kDa molecular ladder band and fragments were incubated with appropriate primary antibodies. X-ray film was used for detection of chemiluminescent signal (the membrane edges are not visible on developed film due to detection of strong and specific signal)**.** Red lines identify cropped areas.

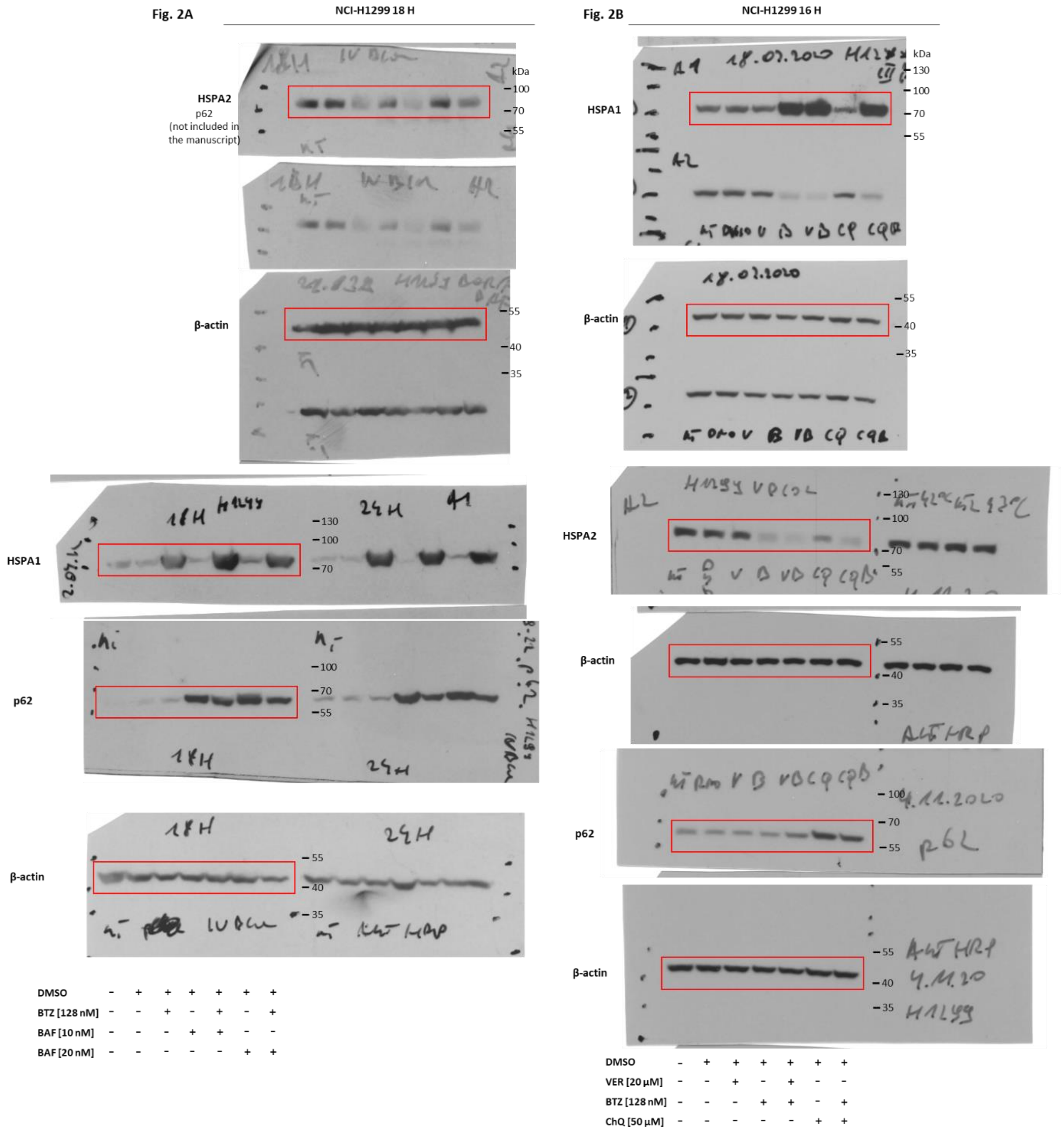

Figure S3. Unprocessed original scans of autoradiograms (immunoblots) included in Fig. 2 A and 2 B. After protein transfer, membranes were cut according to the 55 kDa molecular ladder band and each fragment was incubated with primary antibody. Chemiluminescent signal was detected using X-ray film (the membrane edges are not visible on developed film due to detection of strong and specific signal). Red lines identify cropped areas. In Fig. 2A the same membrane was used for HSPA1 and p62 detection after antibodies stripping in Restore Western Blot Stripping Buffer (Thermo Fisher Scientific, Waltham, MA, USA).

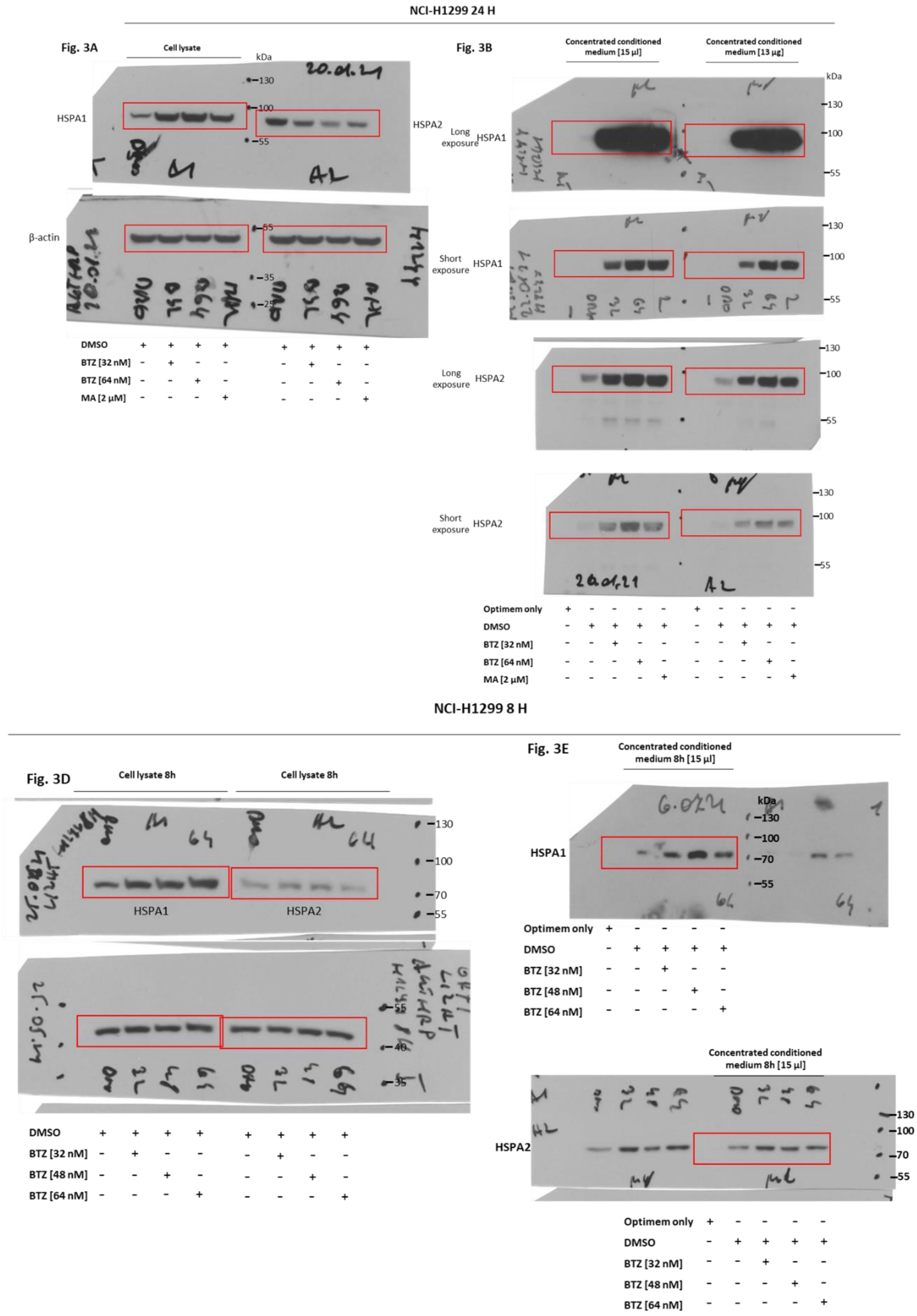

Figure S4. Unprocessed original scans of autoradiograms (immunoblots) included in Fig. 3 A-B and Fig. 3 D-E. After protein transfer, membranes were cut according to the 55 kDa molecular ladder band and were incubated with primary antibodies. Chemiluminescent signal was detected using X-ray film (blot edges are not visible on developed film due to detection of strong and specific signal). Red lines identify cropped areas.

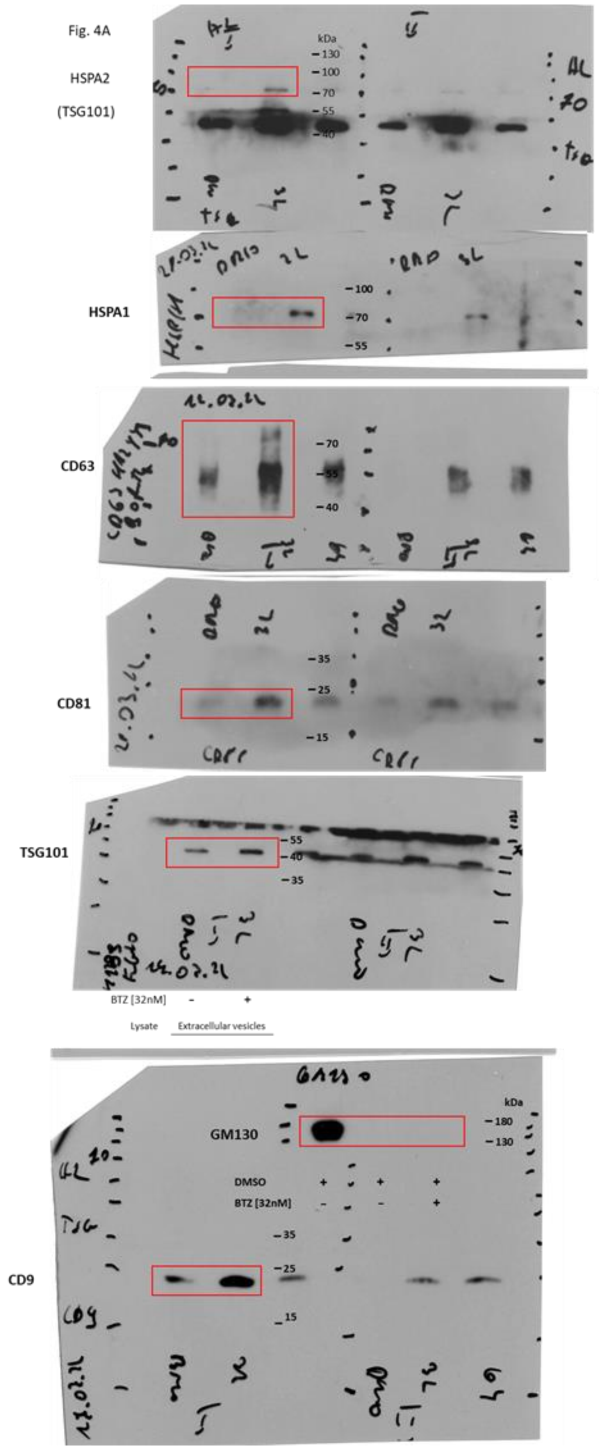

Figure S5. Unprocessed original scans of autoradiograms (immunoblots) included in Fig. 4 A. After protein transfer, membranes were cut according to the proteins' molecular weight and each fragment was incubated with appropriate primary antibody. X-ray film was used for chemiluminescent signal detection (blot edges are not visible on developed film). Red lines identify cropped areas.

HSPA2-knockout in NCI-H1299 cells

Fig. 5A

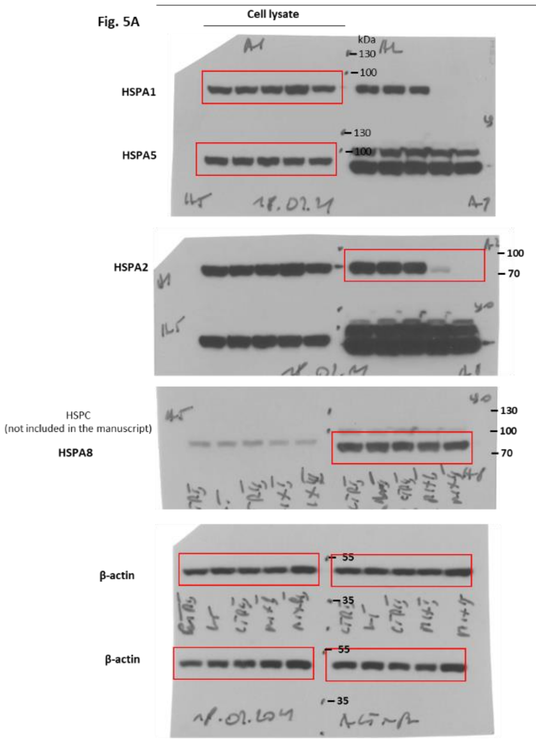

Fig. 5B

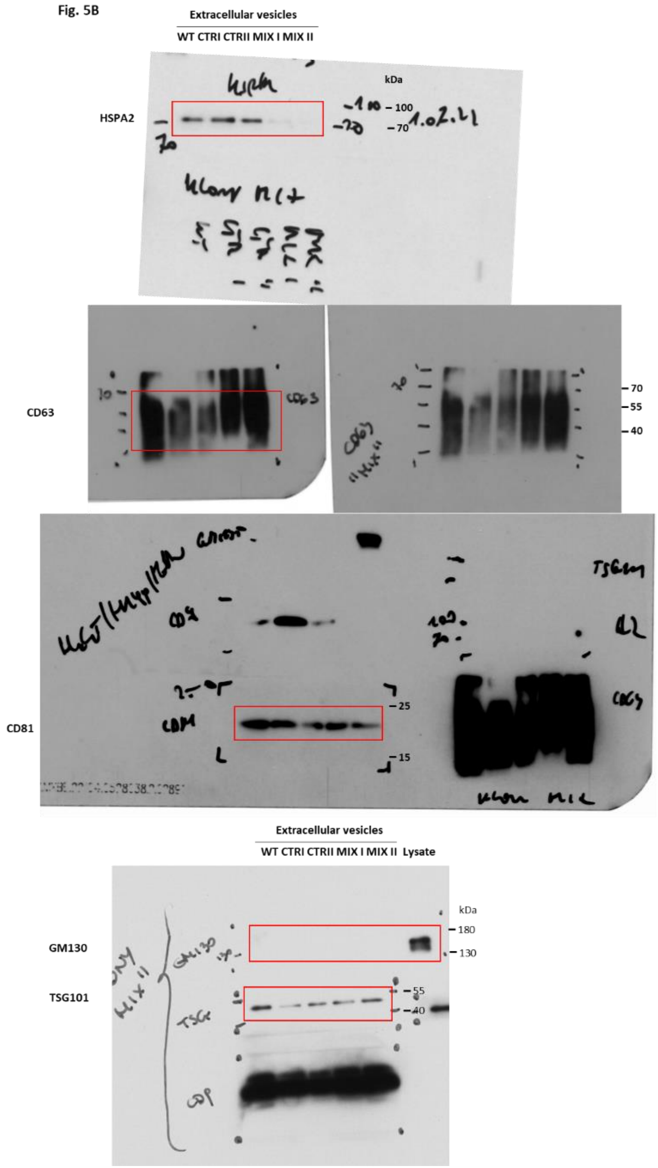

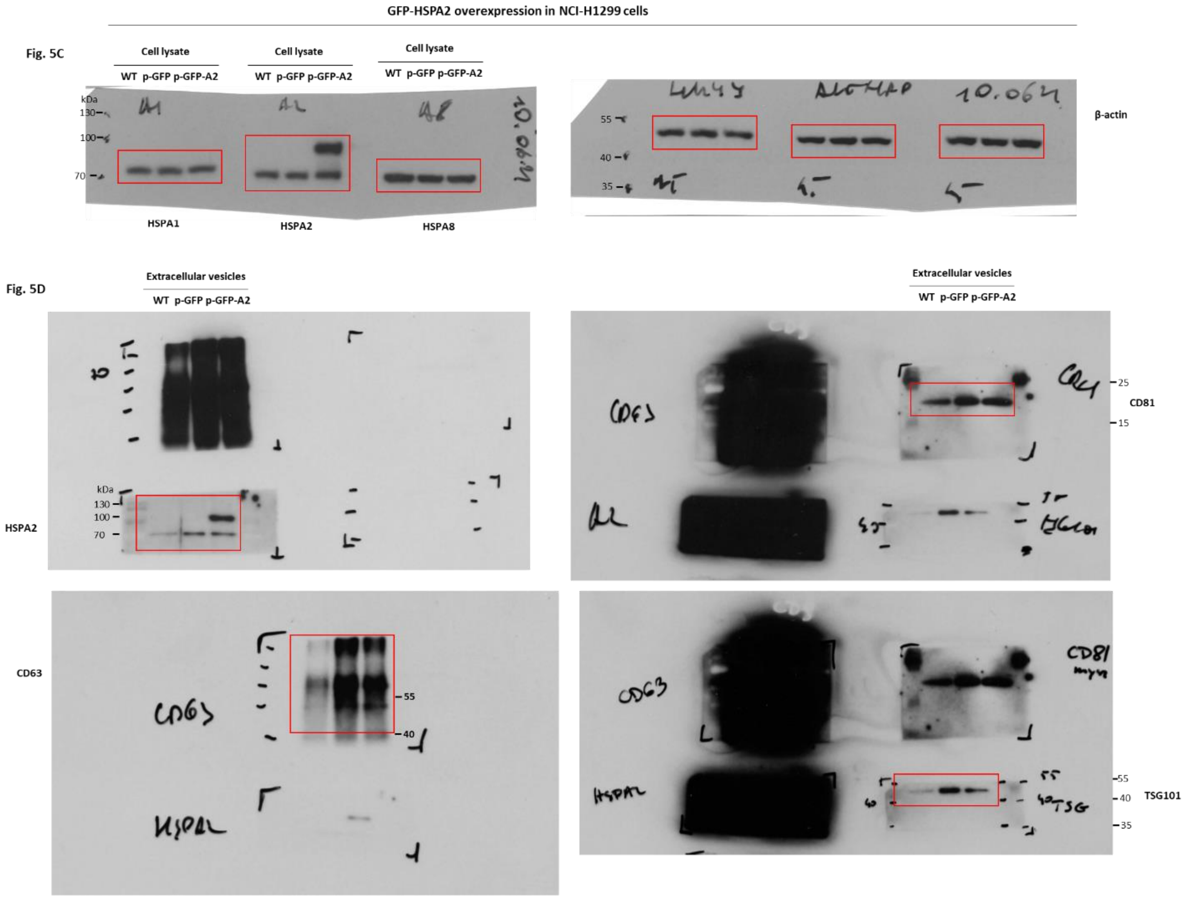

Figure S6. Unprocessed original scans of autoradiograms (immunoblots) included in Fig. 5 A-5D. After protein transfer, membranes were cut into two (or three) fragments according to the proteins’ molecular weight and each fragment was incubated with primary antibody. For chemiluminescent signal detection X-ray film was used. Red lines identify cropped areas.

Fig. 6A

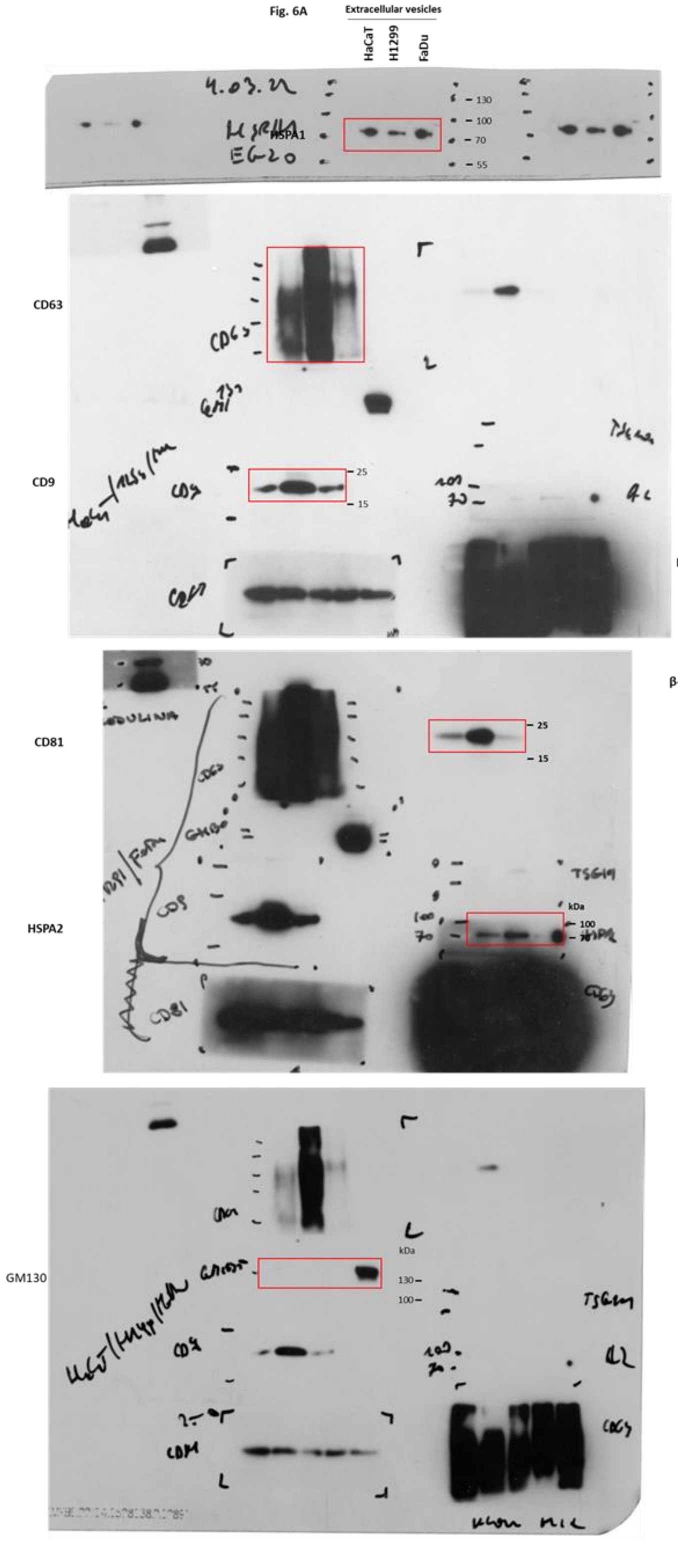

Fig. 6B

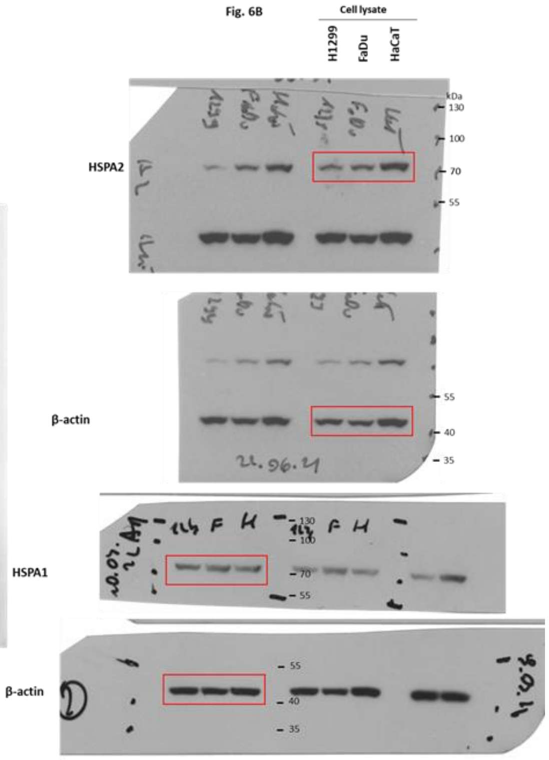

Fig. 6C

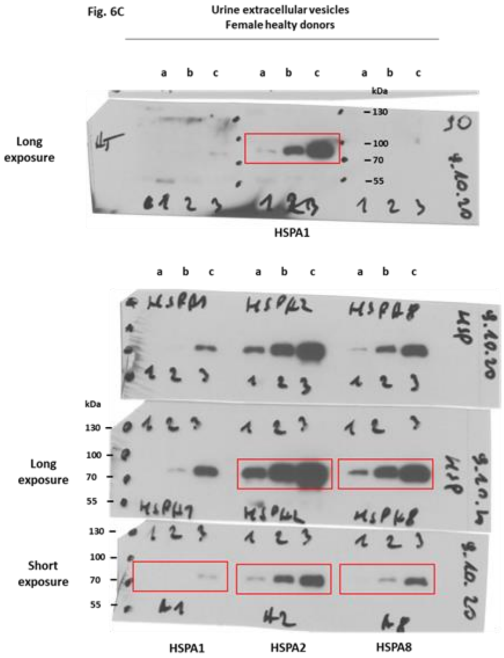

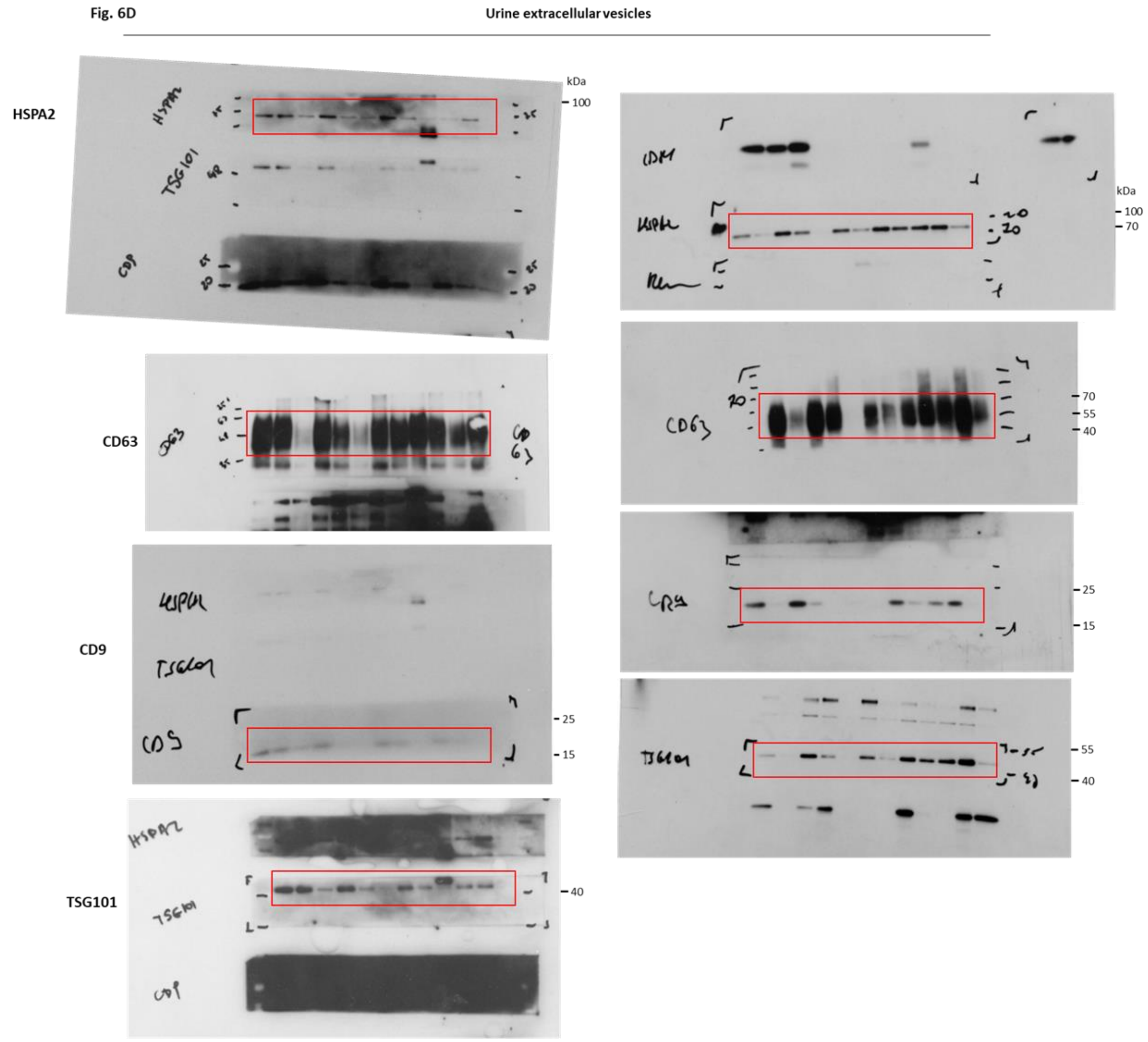

Figure S7. Unprocessed original scans of autoradiograms (immunoblots) included in Fig. 6 A-D. After protein transfer, membranes were cut into two (or more) fragments according to the proteins' molecular weight and each fragment was incubated with primary antibody. For chemiluminescent signal detection X-ray film was used. Red lines identify cropped areas.

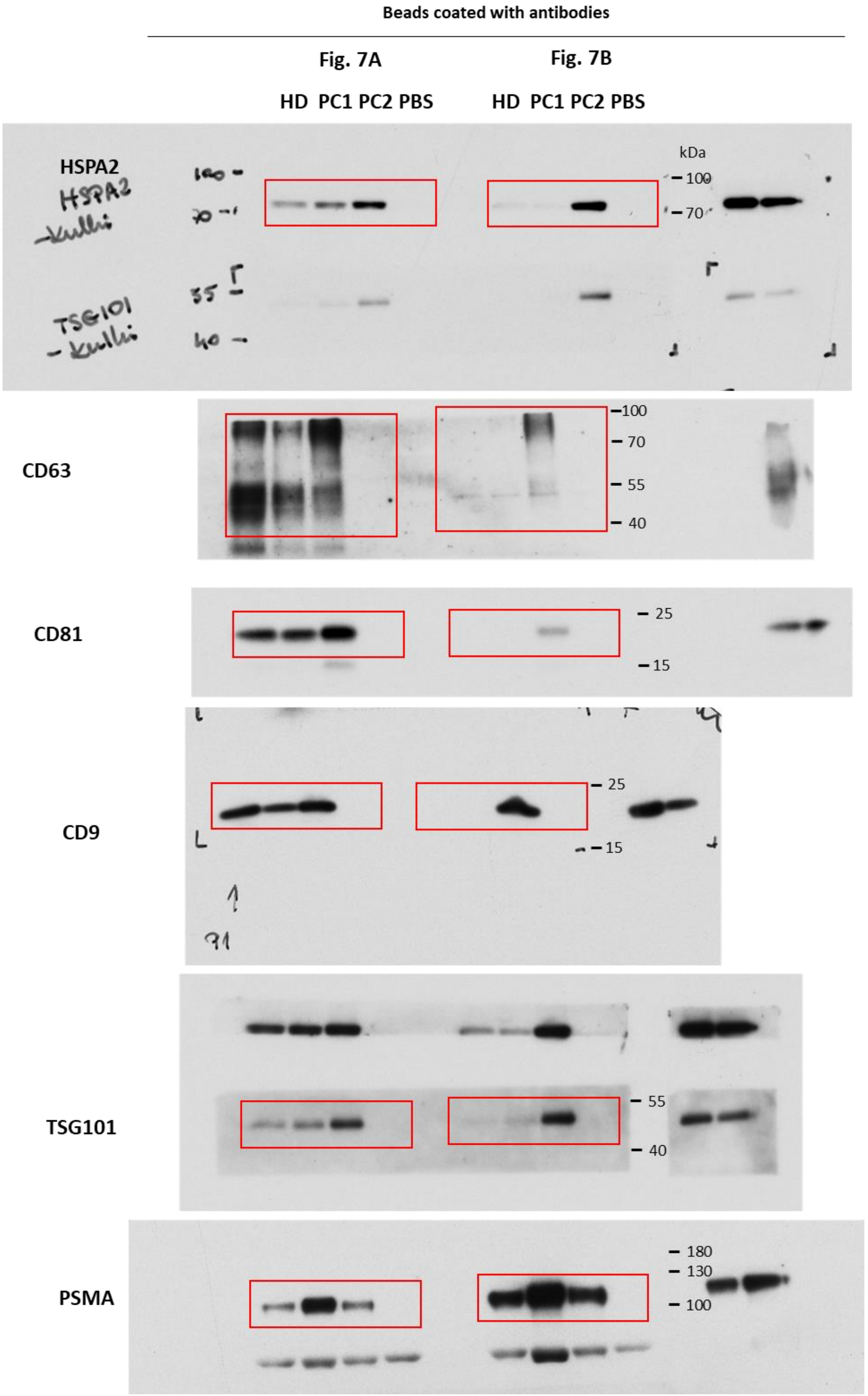

Figure S8. Unprocessed original scans of autoradiograms (immunoblots) included in Fig. 7 A. and 7 B. After protein transfer, were cut into two or more fragments according to the proteins' molecular weight. Each fragment was incubated with primary antibody. X-ray film was used for chemiluminescent signal detection. Red lines identify cropped areas.
